# Computationally designed proteins mimic antibody immune evasion in viral evolution

**DOI:** 10.1016/j.immuni.2025.04.015

**Published:** 2025-05-08

**Authors:** Noor Youssef, Sarah Gurev, Fadi Ghantous, Kelly P. Brock, Javier A. Jaimes, Nicole N. Thadani, Ann Dauphin, Amy C. Sherman, Leonid Yurkovetskiy, Daria Soto, Ralph Estanboulieh, Ben Kotzen, Pascal Notin, Aaron W. Kollasch, Alexander A. Cohen, Sandra E. Dross, Jesse Erasmus, Deborah H. Fuller, Pamela J. Bjorkman, Jacob E. Lemieux, Jeremy Luban, Michael S. Seaman, Debora S. Marks

**Affiliations:** 1 Department of Systems Biology, Harvard Medical School, Boston, MA 02115, USA; 2 Broad Institute of Harvard and MIT, Cambridge, MA 02139, USA; 3 Department of Electrical Engineering and Computer Science, MIT, Cambridge, MA 02139, USA; 4 Center for Virology and Vaccine Research Beth Israel Deaconess Medical Center, Boston, MA 02215, USA; 5 Program in Molecular Medicine, University of Massachusetts Chan Medical School, Worcester, MA 01655, USA; 6 Division of Infectious Diseases, Department of Medicine, Brigham and Women’s Hospital, Boston, MA 02115, USA; 7 Massachusetts General Hospital, Boston, MA 02114, USA; 8 Division of Biology and Biological Engineering, California Institute of Technology, Pasadena, CA 91125, USA; 9 Department of Microbiology, University of Washington, Seattle, WA 98195, USA; 10 National Primate Research Center, Seattle, WA 98109, USA; 11 HDT Bio, Seattle, WA 98109, USA; 12 Massachusetts Consortium on Pathogen Readiness, Boston, MA 02115, USA; 13 Ragon Institute of MGH, MIT, and Harvard, Cambridge, MA 02139, USA; 14 Present address: Kernal Biologics, Cambridge, MA 02142, USA; 15 Present address: Apriori Bio, Boston, MA 02142, USA; 16 Lead contact

## Abstract

Recurrent waves of viral infection necessitate vaccines and therapeutics that remain effective against emerging viruses. Our ability to evaluate interventions is currently limited to assessments against past or circulating variants, which likely differ in their immune escape potential compared with future variants. To address this, we developed EVE-Vax, a computational method for designing antigens that foreshadow immune escape observed in future viral variants. We designed 83 SARS-CoV-2 spike proteins that transduced ACE2-positive cells and displayed neutralization resistance comparable to variants that emerged up to 12 months later in the COVID-19 pandemic. Designed spikes foretold antibody escape from B.1-BA.4/5 bivalent booster sera seen in later variants. The designed constructs also highlighted the increased neutralization breadth elicited by nanoparticle-based, compared with mRNA-based, boosters in non-human primates. Our approach offers targeted panels of synthetic proteins that map the immune landscape for early vaccine and therapeutic evaluation against future viral strains.

## In brief

Rapid viral evolution challenges the efficacy of vaccines and other medical interventions. Our ability to evaluate interventions is currently limited to assessments against past or circulating variants. Youssef et al. developed EVE-Vax, a computational method for designing poly-mutant proteins that foreshadow immune evasion seen in naturally evolving viral variants. Their findings offer an approach for proactive vaccine and therapeutic evaluations.

## Graphical Abstract

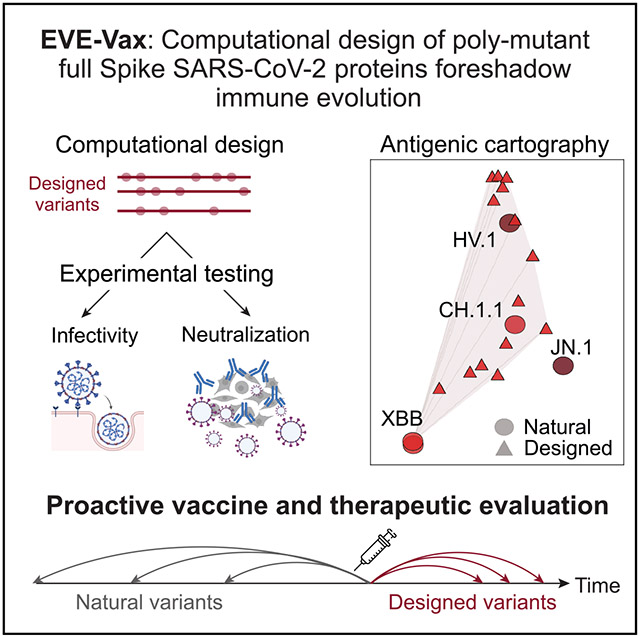

## INTRODUCTION

The emergence of viral variants that evade protective immunity induced by prior infections, vaccines, and therapeutics is a challenge for the control of viral spread. This is exemplified by recurrent breakthrough infections observed with SARS-CoV-2 and influenza. Although SARS-CoV-2 vaccines and therapeutics have mitigated the severity of COVID-19, their efficacy has been progressively undermined by the evolution of new variants. For instance, the U.S. food and drug adminstration (FDA) emergency use approval for the majority of monoclonal antibody therapies has been revoked based on their loss of efficacy against emerging variants of concern (VOCs).^1^ Similarly, first-generation vaccines and boosters exhibit reduced protection against more recent variants,^2,3^ necessitating annual updates to vaccine formulations encoding the latest VOC circulating at the time of vaccine approval. The selected immunogen strain often differs from the dominant variant at the time the vaccine is administered,^4^ leading to lower neutralizing antibody titers.^5^ These limitations highlight the need for strategies that enable proactive, rather than reactive, responses to viral evolution.

Current preclinical evaluations of vaccine and therapeutic efficacy focus primarily on neutralization potency against previous or circulating viral strains or against related viruses, e.g., SARS-like betacoronaviruses (sarbecoviruses).^6-11^ However, neutralizing antibodies that are broadly protective across sarbecoviruses or against circulating SARS-CoV-2 strains do not guarantee protection against emerging variants.^12^ Such evaluations therefore do not address the question of how effectively interventions will protect against future viral evolution. By contrast, predictive frameworks that anticipate immune-evasive mutations could enable the design of viral proteins to proactively assess the breadth and potency of vaccine-elicited and therapeutic antibodies.

Both experimental and computational methods have identified future escape mutations from monoclonal antibodies and polyclonal sera.^2,3,6,11,13-30^ Experimental approaches often utilize deep mutational scanning (DMS) to measure the effect of mutations on neutralization by patient antibodies or sera. However, DMS is typically limited to a subdomain of the antigen, measures the impact of single amino acid mutations or a limited number of combinations of mutations, and relies on patient sera that may not be available early in an outbreak.^2,3,6,11,13-29^ Computational models offer a promising alternative because they bypass these limitations, could be generalizable across viral families, and may be available prior to a viral outbreak. For example, EVEscape, a computational deep learning model, accurately predicted immune-evading mutations across SARS-CoV-2, influenza, HIV, and Lassa virus.^30^ However, although experimental approaches have been used to generate antigens with novel combinations of mutations that evade neutralization, it has yet to be shown that computational methods can similarly generate functional antigens that foreshadow immune escape.

In this work, we address this gap by computationally generating and experimentally testing SARS-CoV-2 spike proteins with novel combinations of mutations that are representative of future antigenic evolution using the EVE-Vax design pipeline. We generated 83 multi-mutant full-length spike constructs across 5 VOC backgrounds (B.1, BA.4/5, BA.2.12.1, BA.2.75, and XBB), which were engineered as single-cycle infection pseudotypes. We assessed neutralization susceptibility against polyclonal immune sera from individuals with diverse exposure histories throughout the COVID-19 pandemic. EVE-Vax-designed spikes replicated immune-escape profiles observed in emerging VOCs demonstrating its potential for mapping the immune landscape and guiding the evaluation and design of medical interventions that are robust to viral evolution.

## RESULTS

A limitation in current vaccine and therapeutic evaluation approaches is that elicited protection is assessed against previous or currently circulating variants of a virus, which does not necessarily reflect efficacy against future viral evolution.^25^ To address this challenge, we developed EVE-Vax, a computational pipeline for generating panels of antigenically diverse proteins, enabling the proactive evaluation of the breadth and future efficacy of vaccines and therapeutics.

**Figure 1.**
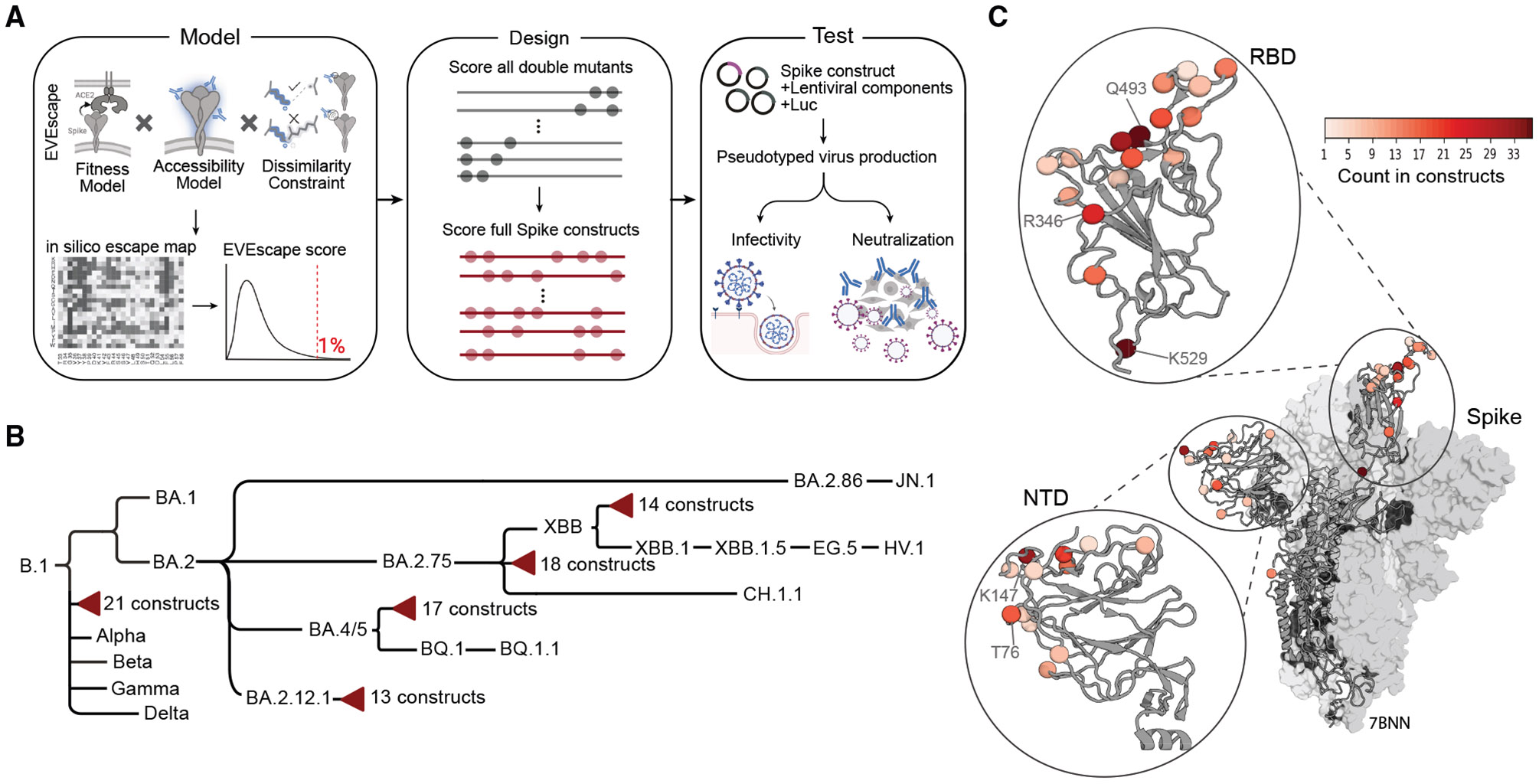
Computationally designed spike proteins allow for a proactive assessment of vaccines and therapeutics (A) Schematic overview of EVE-Vax for designing antigenic proteins. Single mutants within the top 1% of highest-predicted escape scores were combined to generate all possible double mutants. Double mutants were scored and further combined to create multi-mutant constructs. Designed constructs were subsequently evaluated for infectivity and neutralization sensitivity using pseudotyped virus assays. Parts of the figure were created with BioRender. (B) Cladogram depicting VOCs and computationally designed constructs (red triangles). Branch lengths are proportional to the temporal order of variant emergence. (C) Mutations across the 83 designed spike constructs mapped onto a representative 3D structure (PDB: 7BNN). Coloring indicates the frequency with which a given residue was mutated across all designed constructs. See also Figure S1 and Tables S1 and S2.

EVE-Vax uses the EVEscape^30^ framework to score the probability of antibody escape by combining 3 biologically relevant constraints: (1) impact on fitness, (2) accessibility to antibodies, and (3) disruption potential on antibody binding. Briefly, EVE-Vax first combines high-scoring single mutants to evaluate all possible double mutants, which are then further combined to generate multi-mutant full-length spike constructs (Figures 1A and S1; STAR Methods).

We designed 83 multi-mutant spike proteins on 5 different VOC backgrounds: B.1 (wild type variant with D614G mutation), BA.4/5, BA.2.12.1, BA.2.75, and XBB (Figures 1B and S1; Table S1). Constructs contained up to 10 novel combinations of mutations relative to the background VOC and up to 46 mutations relative to the ancestral B.1 strain (Table S2). In total, 37 unique mutations, across 30 positions, were introduced in different combinations and on different spike backgrounds (Figure 1C). Most mutations were located in primary antigenic regions despite no explicit inclusion of this constraint in the EVE-Vax pipeline—17 (57%) of the mutated residues were in the receptor binding domain (RBD) and 12 (40%) were in the N-terminal domain (NTD).

### Computationally designed multi-mutant spike proteins retain infectivity *in vitro*

**Figure 2.**
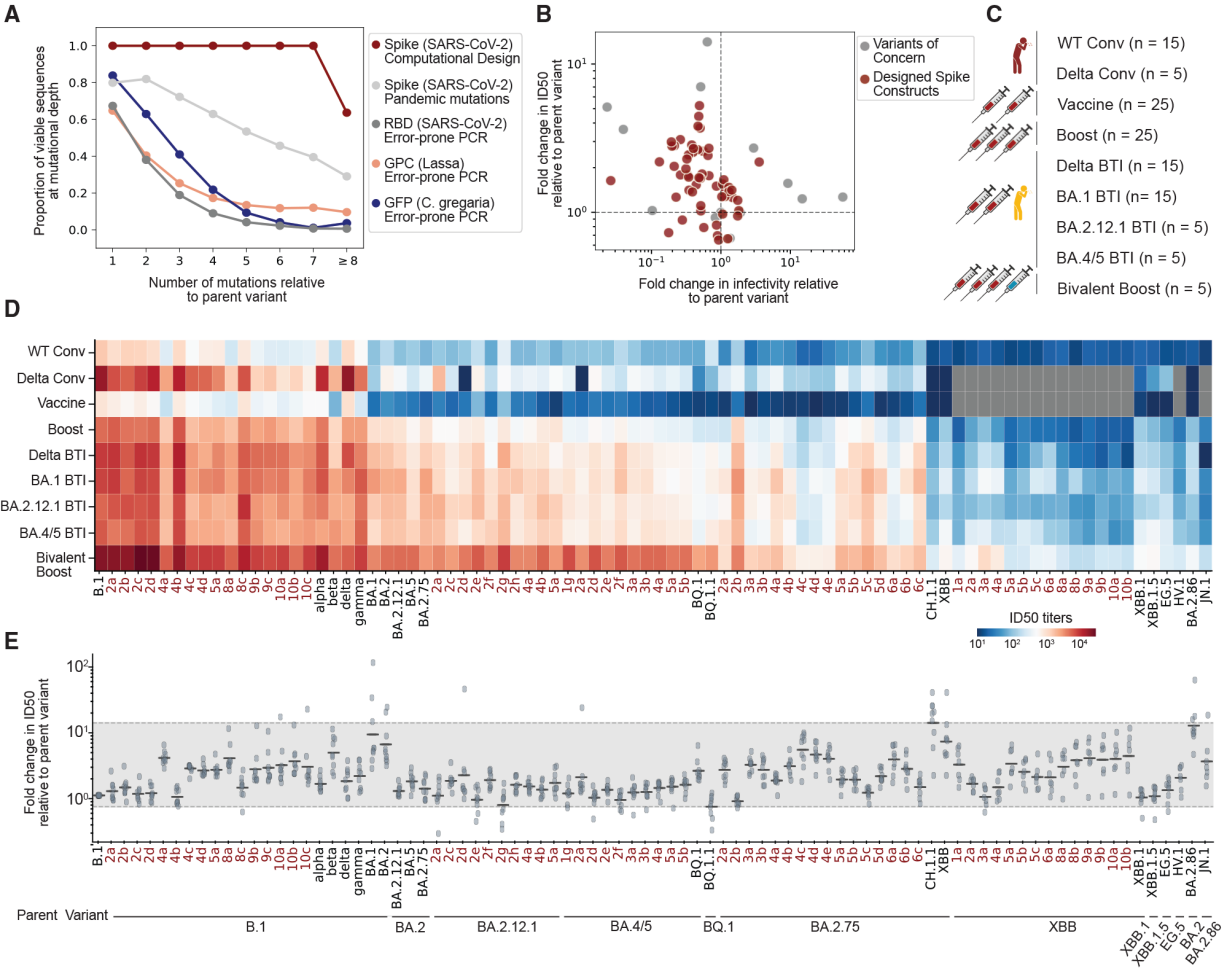
EVE-Vax-designed constructs demonstrate neutralization escape comparable to VOCs (A) Proportion of viable sequences across mutational depths. EVE-Vax-designed constructs exhibit higher viability compared with sequences generated by error-prone PCR within the SARS-CoV-2 RBD (assayed for expression^31^), the Lassa glycoprotein complex (GPC) (assayed for cell entry),^32^ the *Clytia gregaria* green fluorescent protein (GFP, assayed for fluorescence activity^33^), or from combinations of frequently observed pandemic mutations in spike (assayed for infectivity^29^). (B) Relationship between infectivity and neutralization. EVE-Vax-designed constructs and VOCs with higher escape from sera typically showed reduced infectivity relative to their parent variant. (C) Summary of 9 serum panels used for neutralization assays. Panels were from individuals who were convalescent (Conv.), vaccinated, boosted, or experienced breakthrough infection (BTI). A total of 23 serum pools were created, each pooling samples from 5 individuals. (D) Neutralizing ID_50_ titers for VOCs and designed constructs across 9 serum panels. Reported are the geometric mean titers across serum pools within a panel. Gray boxes indicate variants that were not tested in that serum panel. EVE-Vax constructs are highlighted in red text. (E) Fold change in neutralizing ID_50_ titers relative to the parent variant. Points represent the geometric mean fold change for each serum panel, with lines indicating the overall geometric mean across all 9 panels. The gray band represents the range of neutralization sensitivity observed among VOCs. See also Figure S2 and Tables S3 and S4.

Generating multi-mutant proteins is challenging as functionality often decreases with increasing mutational load. For example, in experiments that measured RBD expression using error-prone PCR libraries,^31^ ~47% of all single mutations were expressed in contrast to less than 2% of those with 8 or more mutations (Figure 2A). The reduction in functional sequences with increasing mutational depth is generally observed across viral (Lassa glycoprotein complex, GPC^32^) and non-viral (*Clytia gregaria* green fluorescent protein, GFP^33^) proteins (Figure 2A). Furthermore, full spike proteins containing combinations of commonly observed pandemic mutations, rather than introducing mutations at random using error-prone PCR, resulted in less than 30% of proteins with 8 or more mutations maintaining infectivity.^29^

Among the 83 designed constructs, 90% (75/83) were infectious using replication-incompetent lentiviral-based pseudoviruses in single-round biosafety-level-2 infection assays^34^ (Figures 2A, 2B, and S2; Table S3). The 10% of our designs that were not infectious may be explained post hoc by two observations: (1) 4 of the 8 non-infectious designs contained a triplet of mutations (L452R, F490R, and Q493S) that were closer in the three-dimensional structure than any triplets seen in the pandemic (Figure S2), and (2) the remaining 4 constructs were designed using a model trained exclusively on pre-pandemic sequences and included a mutation (R403T) that likely disrupted ACE2 binding.^35^ Together, these results highlight that the 90% success rate observed in our computationally designed proteins exceeds the expected rates for randomly introduced mutations or combinations of individually non-deleterious mutations and provide insights for refining the EVE-Vax design algorithm.

### Spikes designed on early SARS-CoV-2 variants exhibit neutralization resistance similar to subsequent variants

To evaluate the relevance of the EVE-Vax-designed spike proteins as proxies for future viral variation, we performed pseudovirus neutralization assays using nine panels of human polyclonal serum pools representing diverse SARS-CoV-2 exposure histories: convalescent sera from unvaccinated individuals infected with (1) B.1 or (2) Delta infections; (3) recipients of two primary doses of mRNA vaccines (mRNA-1273 or BNT162b2); (4) recipients of three doses of an mRNA vaccine; (5) vaccinated individuals boosted with BA.4/5 bivalent mRNA vaccines; and vaccinated patients who experienced breakthrough infections (BTIs) with (6) Delta, (7) BA.1, (8) BA.2.12.1, or (9) BA.4/5 variants (Figure 2C; Table S4). Neutralization assays were conducted on pseudoviruses expressing 66 of the designed spike constructs, 20 SARS-CoV-2 variants (B.1, alpha, beta, delta, gamma, BA.1, BA.2, BA.2.12.1, BA.2.75, BA.4/5, BQ.1, BQ.1.1, XBB, XBB.1, XBB.1.5, CH.1.1, EG.5, HV.1, BA.2.86, and JN.1), and the SARS-CoV-1 spike protein (Figure 2D; Table S4).

To characterize the degree of immune evasion observed throughout the pandemic, we compared the half-maximal neutralizing antibody titers (ID_50_) for each variant relative to the parent variant from which it evolved (Figure 2E). As expected, most variants were less susceptible to neutralization (had higher antibody escape) compared with the parental variant that preceded it, with the exception of BQ.1.1, XBB.1, and XBB.1.5 variants, which retained similar titers to their parent variants, BQ.1, XBB, and XBB.1, respectively (*p* > 0.2, Wilcoxon rank-sum test). The XBB (7.2-fold, *p* < 0.01, Wilcoxon rank-sum test) and CH.1.1 (14.2-fold, *p* < 0.01, Wilcoxon rank-sum test) variants displayed the highest neutralization resistance relative to their parent variant BA.2.75. On average, emerging variants exhibited a 3.9-fold reduction in geometric mean ID_50_ titers relative to their parent variant and a range from 0.67 to 14.2 (gray region, Figure 2E). Variants with higher antibody escape generally exhibited reduced infectivity compared with their parent variants (Figure 2B).

Pseudoviruses expressing designed spikes had comparable neutralization titers as SARS-CoV-2 variants that evolved throughout the pandemic. On average, EVE-vax-designed spikes had a 1.9-fold reduction (range of 0.5 to 5.31) in geometric mean ID_50_ titer compared with their relative parent variant (Figure 2E). More specifically, designs on specific backgrounds had similar neutralization resistance as SARS-CoV-2 variants evolved from those same backgrounds. For instance, the B.1-4a-designed construct (B.1 + K147N + S494R + F490R + R683N) had a 3.9-fold reduction in geometric mean ID_50_ titer, exceeding the neutralization resistance of Alpha, Delta, and Gamma (1.5-, 1.7-, and 2.0-fold reduction relative to B.1, respectively) and comparable to Beta (4.8-fold reduction relative to B.1). Similarly, the BA.4/5-2a construct (BA.4/5 + R346T + S494R) exhibited a 1.9-fold reduction in geometric mean ID_50_ titers relative to BA.4/5, comparable to BQ.1 (2.4-fold reduction). The BA.2.75-4c design (BA.2.75 + G339D + L452R + Q493R + K529L) showed a 5.3-fold reduction in geometric mean ID_50_ titer relative to BA.2.75, below the relative neutralization resistance of XBB (7.2-fold). Neutralization profiles of EVE-vax designed constructs on the XBB background remain consistent with the most recent SARS-CoV-2 variants (JN.1.7.1, XDV, KQ.1, KP.1.1, KP.2, KP.1.1.1, KP.3, KP.3.1.1, and LB.1), which emerged nearly two years after the designs (Figure S3). Together, these results demonstrate that EVE-Vax designed constructs exhibit a similar antibody escape capacity as variants that have naturally evolved under immune pressure and can therefore serve as useful proxies for future SARS-CoV-2 evolution.

### Designed variants foreshadow antigenic evolution

**Figure 3.**
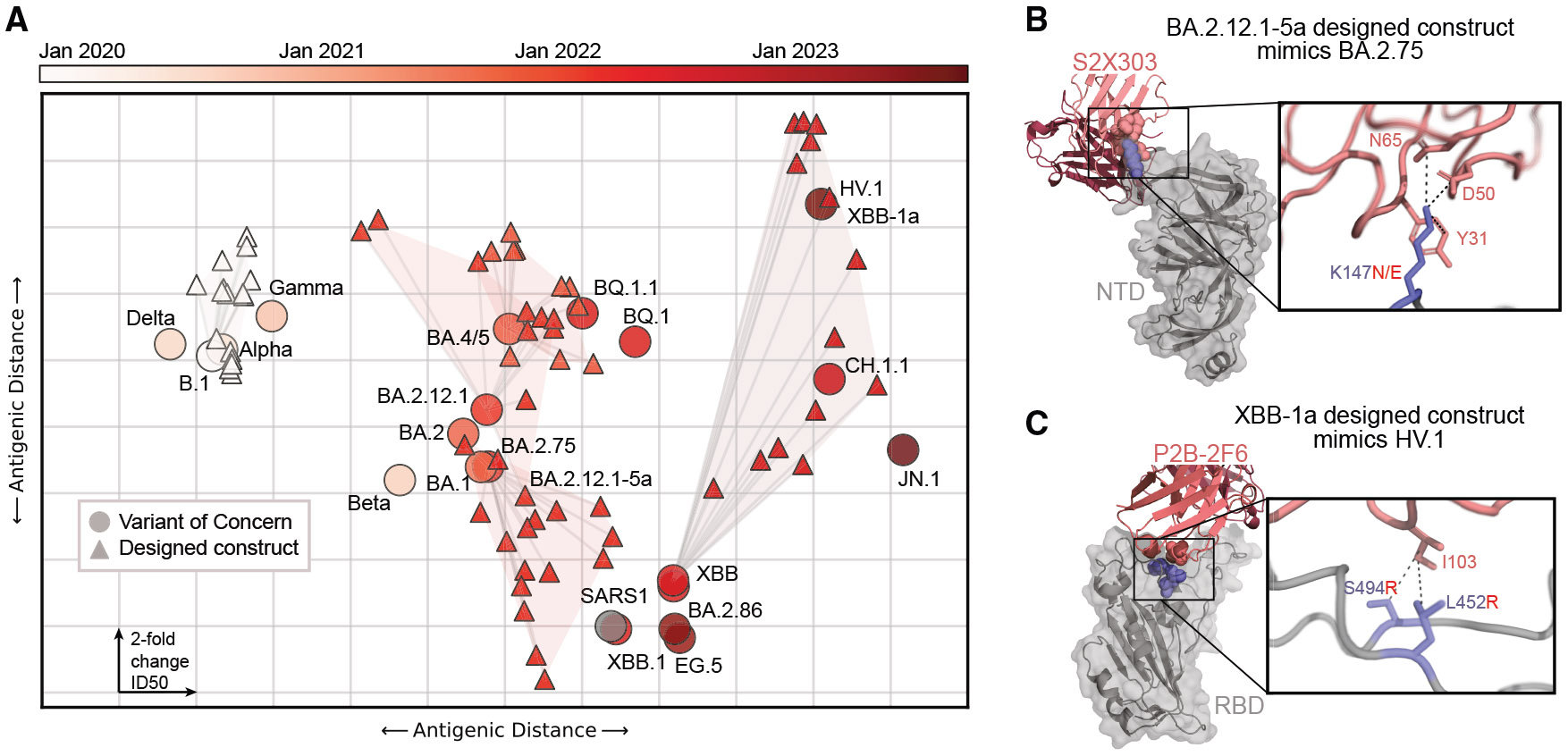
EVE-Vax-designed constructs foreshadow future SARS-CoV-2 immune-escape evolution (A) Antigenic cartography depicts a two-dimensional representation of the antigenic distance based on neutralization by 23 serum pools. Each grid box corresponds to a 2-fold change in neutralizing ID_50_ titers. Variants (circles) are colored according to their month of emergence since the onset of the pandemic. Designed constructs (triangles) on earlier variants foreshadow the antigenicity of later pandemic variants, as indicated by proximity in the antigenic map. (B) Designed construct BA.2.12.1-5a mimics the neutralizability of later pandemic variant BA.2.75. Both contain a mutation at K147 (N in the design, E in the later variant). The impact of K147 mutations can be seen for NTD antibody S2X303, with interactions to N65, D50, and Y31 on the antibody (PDB: 7SOF). (C) Many XBB designs containing L452R (XBB-8a,9a,9b,10a,10b) or S494R (XBB-1a) closely resemble the neutralization profile of the later HV.1 variant, which also harbors L452R. Arginine mutations in either S494 or L452 result in escape from RBD class 2 antibody P2B-2F6^2,37^ (PDB: 8DCC). See also Figure S3 and Table S4.

To investigate whether EVE-Vax designed constructs recapitulate the antigenic evolution observed during the pandemic, we constructed antigenic maps where the distance between any pair of variants reflects the similarity in their neutralization profiles across diverse serum pools (Figures 3A and S3). Constructs designed on earlier variants exhibited an antigenic resemblance to variants that emerged later in the pandemic (Figure 3A). The BA.2.12.1-5a designed construct mimics the neutralizability of later pandemic variant BA.2.75 with both variants mutating residue K147 (N in the design, E in the later variant). The functional impact of K147 mutations can be seen for the NTD antibody S2X303, whose epitope partially overlaps the NTD antigenic supersite^36^ (Figure 3B). XBB designs that contained L452R (XBB-8a,9a,9b,10a,10b) or S494R (XBB-1a) closely matched the neutralization profile of HV.1, which also contained L452R, with notable escape from BA.1 BTI sera compared with other serum panels (Table S4). The impacts of these mutations can be seen in RBD class 2 antibody P2B-2F6,^37^ where mutation to an arginine of either S494 or L452 (both contacting I103 on the antibody) results in escape^2^ (Figure 3C). These observations align with the hypothesis that later Omicron subvariants evolved L452R to enhance immune evasion following BA.1 infection.^3^ These trends were consistent across other rounds of design: constructs designed on BA.2.75 were antigenically similar to the XBB lineages that emerged nine months later, constructs designed on the XBB variant exhibited antigenic responses akin to the CH.1.1 and HV.1 variants that emerged three and 12 months later, constructs based on BA.4/5 anticipated BQ.1.1, and designs on B.1 presaged the alpha and gamma variants (Figure 3A, shaded area). These findings underscore the capability of EVE-Vax designs to recapitulate antigenic profiles akin to those of future variants using only data available at the time of VOC emergence.

### Evaluating neutralizing antibody responses elicited by mRNA vaccines

**Figure 4.**
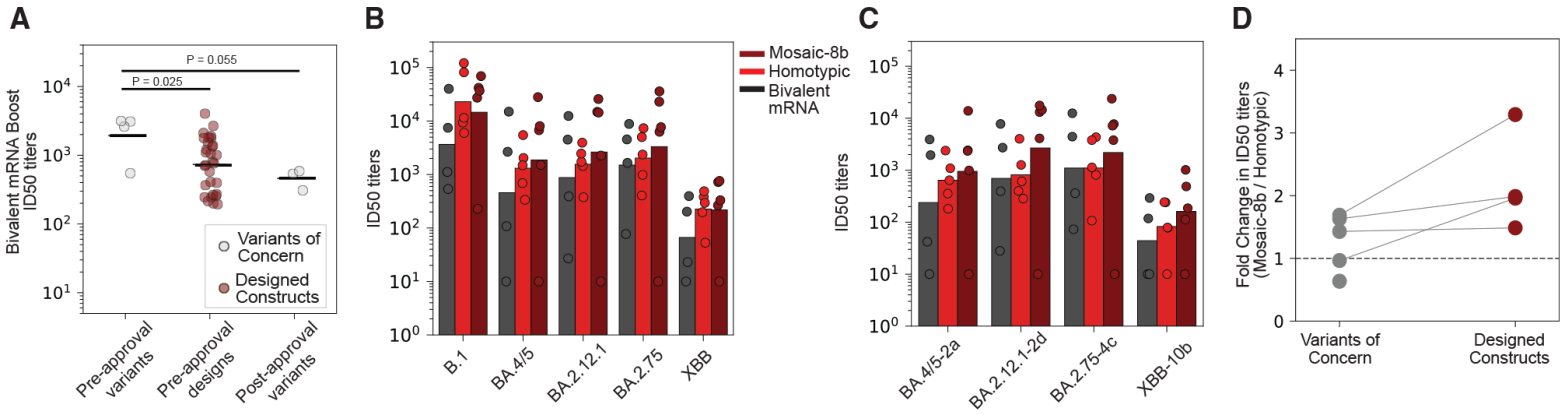
Evaluating the breadth and potency of vaccines using designed spike constructs (A) Neutralizing ID_50_ titers against sera from individuals who received a BA.4/5 bivalent booster vaccination after 3 doses of an mRNA vaccine. Lines indicate geometric mean across variants or designs. The BA.4/5 bivalent booster vaccine was evaluated on its ability to neutralize variants circulating in the 4 months prior to the creation of the booster vaccines (BA.2.75, BQ.1, BQ.1.1, and XBB). Designed spike constructs on the background of variants circulating prior to the vaccine foreshadowed the antibody escape observed against variants that emerged following the bivalent booster approval (XBB.1, XBB.1.5, and CH.1.1). Statistical significance was assessed using the Wilcoxon rank-sum test. (B) Neutralizing potency of sera collected from non-human primates primed with a mixture of nucleic acid vaccines and boosted with either a bivalent mRNA (*n* = 4), a homotypic nanoparticle (*n* = 5), or a mosaic 8b nanoparticle (*n* = 5). Bar charts represent the geometric mean ID_50_ titers for each variant. (C) Bar charts represent the geometric mean ID_50_ titers for each designed construct. Designed constructs highlight the improvement of protection from antibodies elicited by the mosaic-8b booster compared with the homotypic nanoparticle. (D) Neutralization sensitivity of a given variant by the mosaic-8b boost sera relative to the homotypic boost sera. Higher values indicate the mosaic-8b vaccine better neutralizes a given variant. Lines connect designed constructs to their parent variants. See also Table S4.

To demonstrate the utility of our approach in the proactive evaluation of vaccine efficacy, we retrospectively used the designed constructs to evaluate the B.1-BA.4/5 bivalent booster vaccine. Considering variants circulating in the four months preceding the bivalent booster campaign (BA.2.75, BQ.1, BQ.1.1, and XBB), the geometric mean ID_50_ titers were 1,931 for serum panels representative of bivalent boosting (four shots; Figure 4A; Table S1 and S4). The high titers are indicative of adequate protection against these prior variants. However, constructs designed on BA.2.75 and XBB demonstrated a range of antibody escape, with ID_50_ titers ranging from 193 to 4,029. The broad range of titers, specifically the low titers, were reflective of the escape potential of variants that evolved post booster vaccine implementation—the subsequent variants, XBB.1, XBB.1.5, and CH.1.1, had ID_50_ titers of 538, 587, and 308, respectively (Figure 4A). Moreover, 8 XBB-based designs displayed lower ID_50_ titers than CH.1.1, demonstrating the potential for further escape from neutralizing antibodies elicited by bivalent booster vaccines. These results highlight the utility of EVE-Vax designs for early vaccine evaluation.

### Evaluating neutralizing antibody responses elicited by nanoparticle vaccines

Cohen et al.^8,9^ recently developed two RBD nanoparticle vaccines: mosaic-8b nanoparticles displaying RBDs from eight different sarbecoviruses and a homotypic nanoparticle displaying the RBD from the Beta SARS-CoV-2 variant. Both nanoparticle vaccines elicited neutralizing antibodies against SARS-CoV-2 variants when tested in mouse and non-human primate (NHP) animal models.^8,9^ Fourteen NHPs were primed with a mixture of nucleotide vaccines and boosted with either a bivalent mRNA vaccine (*n* = 4), a mosaic-8b nanoparticle (*n* = 5), or a homotypic nanoparticle (*n* = 5) vaccine.^38^ To evaluate the neutralizing potential of these booster vaccines against future SARS-CoV-2 evolution, we examined differences in neutralization activity across five SARS-CoV-2 variants (B.1, BA.2.12.1, BA.2.75, BA.4/5, and XBB), as well as four designed spike constructs (BA.5-2a, BA.2.12.1-2d, BA.2.75-4c, and XBB-10b; Table S4).

Sera from bivalent mRNA-boosted NHPs demonstrated the lowest neutralization titers against the tested SARS-CoV-2 variants (mean titer of 678) compared with sera from NHPs, which were boosted with the homotypic nanoparticle (1,843, *P* = 0.166 Wilcoxon rank-sum test) or the mosaic-8b nanoparticle (2,199, *p* = 0.060, Wilcoxon rank-sum test, Figure 4B). Neutralizing titers were consistently lower against the EVE-Vax designed constructs compared with the SARS-CoV-2 variants, with on average a 2.3, 4.0, and 2.3-fold decrease in the bivalent mRNA, homotypic, and mosaic-8b sera, respectively (Figure 4C). Although the neutralization titers among the natural SARS-CoV-2 variants were comparable between the mosaic-8b and homotypic-boosted NHPs, the designed constructs showcase the improved performance of the mosaic-8b nanoparticle booster vaccine (Figure 4D). Neutralizing titers against the designed constructs BA.2.12.1-2d, BA.2.75-4c, BA.5-2a, and XBB-10b were 3.3, 2.0, 1.5, and 2.0-fold higher in sera from mosaic-8b boosted NHPs compared with homotypic boosted animals. Overall, the results suggest that nanoparticle booster vaccines lead to higher neutralizing titers against future SARS-CoV-2 variants as compared with bivalent mRNA booster vaccines and that the mosaic-8b elicits more cross-reactive antibodies compared with the homotypic nanoparticle.

### EVE-Vax designs have comparable escape to assay-derived designs

**Figure 5.**
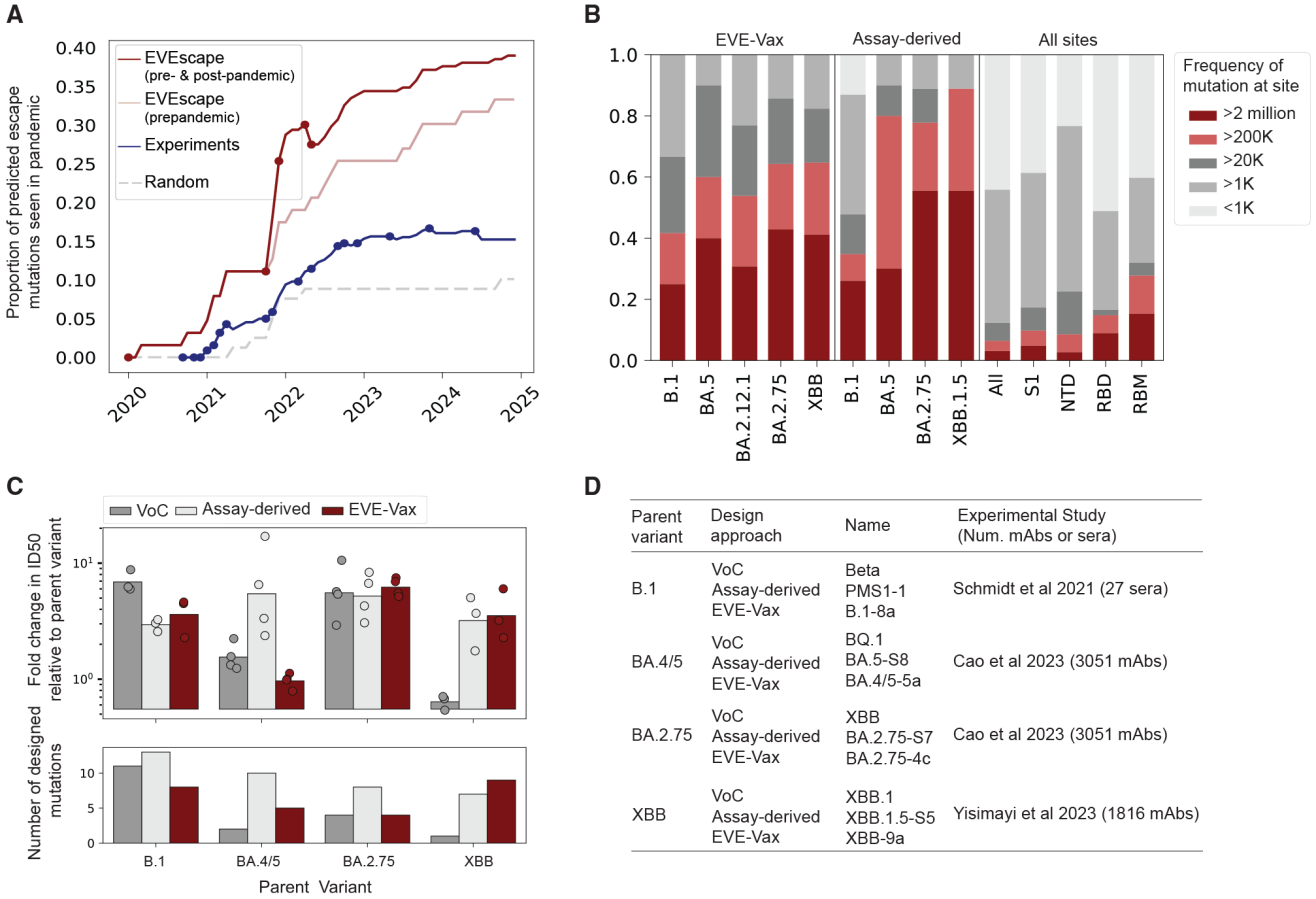
EVE-Vax constructs have comparable polyclonal escape to constructs designed using pandemic sera and antibodies (A) Proportion of escape mutations seen in over 1,000 strains in Global Initiative on Sharing All Influenza Data (GISAID), as predicted by EVEscape (with and without SARS-CoV-2 sequences), high-throughput DMS experiments across 20 studies (Table S5), or randomly selected RBD mutations. Predicted escape mutations were defined as nonsynonymous, single-nucleotide RBD mutations in the top 5% of escape scores for computational and experimental methods (top 1% and 10% in Figure S4). Points indicate updates to the set of escape mutations based on the availability of a more recent DMS data (blue) or model (red, which includes SARS-CoV-2 sequences available prior to that date). Predictions for the full spike protein (computational) and for each independent experiment and model are provided in Figure S4. (B) Proportion of designed sites mutated at different frequencies in GISAID, categorized by computational or experimental design rounds or by spike regions. Designed mutations are enriched at high-frequency sites compared with immunodominant spike regions (e.g., RBD or NTD). Spike regions analyzed: all (1–1,273), S1 (1–686), NTD (13–305), RBD (319–541), and RBM (437–508). (C) Fold change in ID_50_ titers of assay-derived constructs, EVE-Vax constructs, or descendant VOC relative to the respective parent variant. The construct with the highest neutralization escape was selected for each round of computational and assay-derived designs. Individual points represent fold change for matched sera pool between computational and experimental studies; bars indicate geometric mean fold reduction. (D) Table of selected EVE-Vax construct, assay-derived construct, or descendant VOC for each parent variant. For experimental and computational methods, the construct with the highest average escape per round is reported. RBD, receptor-binding domain; NTD, N-terminal domain; RBM, receptor-binding motif. See also Figures S4 and S5 and Table S5.

Lastly, we compared the ability of computational and experimental methods to forecast pandemic mutations and generate constructs that foreshadow immune escape. Prior work demonstrated that EVEscape, trained on data available prior to the onset of the COVID-19 pandemic, more accurately predicted escape mutations observed during the pandemic than the earliest mutational scanning experiments.^30^ To date, 33% of escape mutations predicted by *pre-pandemic* EVEscape (mutations in the top 5% of scores) were observed in over 1,000 viral strains during the pandemic. In contrast, only 15% of escape mutations predicted by early mutational scanning experiments met this threshold (Figures 5A and S4; Table S5). Updating the computational models with SARS-CoV-2 sequences available through July 2022 (models developed in this study) increased the proportion of observed escape mutations to ~40% (85 of the 218 computationally predicted escape mutations appeared in more than 1,000 sequences). By comparison, updating experimental datasets to reflect contemporary immune pressures by including escape mutations from 20 high-throughput experimental studies^2,3,6,11,13-29^ resulted in ~15% of escape mutations being observed during the pandemic (64 of the 420 experimentally predicted escape mutations).

We compare the ability of EVE-Vax constructs versus “assay-derived” constructs to foreshadow immune escape (assay-derived constructs refer to previously published constructs that were developed using experimental methods). We compared our B.1 designs to PMS1-1 and PMSD4 from Schmidt et al.,^26^ which included 13 mutations in each construct selected based on plasma neutralization sensitivity and distribution across the spike protein (Figure S5). For BA.2.75 and BA.4/5 backgrounds, we compared our constructs to designs from Cao et al.,^22^ where RBD mutations were selected based on criteria derived from deep mutational scans assaying neutralization resistance against over three thousand BA.2-neutralizing antibodies (Figures S5). We compare our XBB designs to the closest available experimental designs from Yisimayi et al.,^23^ who designed XBB.1.5 RBD constructs using the same criteria as Cao et al.^22^ but measured neutralizing activity across 1,816 RBD-targeting antibodies (Figure S5).

Both computational and experimental approaches identified positions that were frequently mutated during the pandemic at greater rates than random selection of mutations in immunodominant regions of spike (Figures 5B and S5). Additionally, EVE-Vax constructs had comparable escape from polyclonal sera to assay-derived constructs while incorporating fewer mutations per spike protein (*p* > 0.05, Mann-Whitney rank-sum test, Bonferroni correction; Figures 5C and 5D). Furthermore, the majority of the escape mutations included in assay-derived designs were high scoring in the computational model scores (Figure S5), highlighting that these mutations could have been identified computationally if the threshold used for identifying escape mutations was lowered in the EVE-Vax pipeline. Conversely, the mutations in our EVE-Vax constructs would not have been identified from the experimental data (Figure S5). These findings indicate that EVE-Vax provides an alternative to high-throughput experimental methods for designing antigenic proteins that mimic immune-escape evolution.

## DISCUSSION

Driven by the need for vaccines and therapeutics that maintain efficacy against viral evolution, hundreds of vaccine candidates are currently undergoing preclinical or clinical evaluations.^39^ However, it remains unclear the extent to which these candidate vaccines will effectively combat future viral variants. To date, neutralization potential of SARS-CoV-2 booster vaccines,^40^ pan-variant therapeutics,^6,41^ and other antivirals^42^ have been evaluated against previous or contemporary SARS-CoV-2 variants or against distantly related sarbecoviruses.^8,9^ Such approaches are limited to retrospectively testing efficacy against emerging pandemic variants or throughout evolution, which do not guarantee effectiveness against future variants.^12^ We present a vaccine and therapeutic evaluation approach that addresses these issues by designing panels of diverse antigens that can be used, in safe non-replicative pseudotype assays, to assess the breadth and potency of candidate vaccines and therapeutics to neutralize future viral variants.

We highlight the utility of this approach through analyses of the bivalent mRNA booster vaccine and pan-sarbecovirus nanoparticle-based vaccines. We demonstrate that the immune escape observed by SARS-CoV-2 variants that emerged following the bivalent booster vaccination campaign, such as XBB and CH.1.1, could have been anticipated at the time that booster vaccines were implemented. Additionally, EVE-Vax-designed constructs demonstrated the potential for further immune escape, which has since been realized by JN.1 and other variants in the BA.2.86 lineage that continue to emerge. Alternatively, nanoparticle-based vaccines offer a promising vaccine modality that has the potential to elicit broadly neutralizing antibodies. The conceptual premise posits that vaccines designed to counteract sarbecoviruses, with broad taxonomic distances, would elicit antibodies targeting conserved regions, thus decreasing the likelihood of escape under immune pressures. Supporting the advantage of the mosaic nanoparticle vaccine, we find higher neutralizing titers against our EVE-Vax constructs compared with the homotypic nanoparticle or mRNA vaccines. Taken together, these findings suggest that, when evaluating vaccines against a panel of designed variants, the range of neutralizing titers, especially the lowest observed values, may signal future escape potential.

A key advantage of the computational approach is the ability to predict escape mutations independent of antibody or sera availability, enabling proactive design efforts before the emergence of novel variants or spillover events. Furthermore, the approach is generalizable across viral families and is likely to be of relevance for mapping the immune landscape of key antigenic proteins and developing countermeasures against highly diverse or poorly characterized viruses. For example, addressing the high diversity of Lassa virus strains presents a significant challenge for vaccine development. EVE-Vax can be used to generate panels of diverse strains to assess the cross-protection potential of vaccines currently in clinical and preclinical trials.^43^ By leveraging non-replicative pseudovirus assays, even biosafety-level 4 pathogens can be evaluated under safer conditions, consistent with established practices.^11,24^ In addition to generating panels of synthetic proteins for vaccine evaluation, evidence presented in this paper suggests that the EVE-Vax pipeline could more directly inform vaccine design by identifying epitopes likely to escape prior vaccine-induced or therapeutic antibodies.

### Limitations of the study

The extent to which the method presented here is generalizable to other viral antigens will depend on many factors—not least, whether the available evolutionary sequence record is sufficient for a computational approach to learn functional constraints. Heuristics for determining the number and diversity of sequences needed to accurately predicti functional constraints have been explored to a limited extent.^44^ More quantitative methods are needed, especially those tailored to viruses, as many viruses with a high pandemic and spillover risk remain understudied and undersequenced. Although the promise of protein language models, trained on a broad set of proteins, may mitigate some of these limitations, their applicability for designing *de novo* proteins for vaccine evaluation and design remains to be explored. Another limitation is the focus in this study on antibody neutralization. Although important for initial clinical efficacy,^45^ other components of the immune response, such as T-cell-mediated immunity, are often essential for long-term protection. Lastly, forecasting future variants, whether experimental or computational, could, in theory, be misused; however, these risks can be minimized by the responsible development and sharing of such information.^46^

## STAR★METHODS

### EXPERIMENTAL MODEL AND STUDY PARTICIPANT DETAILS

#### Cell lines

HEK293T cells (derived from HEK293 Homo sapiens, female, embryonic kidney cells) used here were obtained from the American Type Culture Collection or ATCC (CRL-3216 for 293T or CRL-11268 for 293T/17). HEK293T cells with stably expressed human ACE2 were obtained from the same cell clone previously described by Mou et al.^53^ and were kindly provided by Drs. Michael Farzan and Huihui Ma (The Scripps Research Institute). HEK293T cells were transduced with vesicular stomatitis virus (VSV) G protein-pseudotyped murine leukemia viruses to stably express the human ACE2 cell surface receptor protein. All cells were cultured in humidified incubators with 5% CO2 at 37° C, and monitored for mycoplasma contamination using the Mycoplasma Detection kit (Lonza LT07-318), were cultured in DMEM supplemented with 10% heat-inactivated FBS, 1 mM sodium pyruvate, 20 mM GlutaMAX, 1 × MEM non-essential amino acids, and 25 mM HEPES, pH 7.2. HEK293T cells with stably expression of the human angiotensin converting enzyme 2 (ACE2) and the transmembrane serine protease 2 (TMPRSS2) (HEK293T-ACE2-TMPRSS2 cells) were developed by the Luban laboratory.^34^

#### SARS-CoV-2 convalescent and vaccine human sera

We analyzed 115 serum samples collected from individuals who were either convalescent, vaccinated, boosted, or experienced breakthrough infection (Table S4). Informed consent was obtained from participants in the Post-vaccination Viral Characteristics Study (POSITIVES) study.^47-49^ Serum samples were acquired approximately 2-4 weeks after vaccination or convalescence. For patients experiencing breakthrough infection, whole genome sequencing was performed on a nasal swab collected at the time of diagnosis to confirm infecting SARS-CoV-2 variant.

In order to maximize the number of designed spike variant pseudoviruses that can be tested against the same immune serum panels, a total of 23 serum pools were created by grouping 5 individual patient samples per pool. The pooled sera are representative of the different populations that have existed throughout the pandemic: natural infection, primary vaccination, boosted, bivalent boosted, and breakthrough infection. When sufficient samples were available, individual serum samples were pooled based on neutralization titers resulting in low, medium, and high neutralization pools.

#### Sarbecovirus non-human primate vaccine sera

Fourteen NHPs (5-year-old male and female cynomolgus macaques of Mauritian origin) received four doses of DNA or repRNA SARS-CoV-2 vaccines 64 and 30 weeks prior to boosting with either a BA.1 bivalent mRNA vaccine (n = 4), a mosaic-8b sarbecovirus RBD nanoparticle (n = 5), or a homotypic sarbecovirus RBD nanoparticle (n = 5) vaccine.^38^ Samples were collected 2-weeks post booster. The mixed immune history in these groups of pre-vaccinated NHPs is representative of a complex immune history in people who have been vaccinated and/or infected multiple times.

### METHOD DETAILS

#### Multiple Sequence Alignments for fitness models

We designed spike constructs on the backgrounds of five VOCs: the canonical wildtype SARS-CoV-2 spike protein (Uniprot ID: P0DTC2) with D614G mutation (B.1), and the BA.4/5, BA.2.12.1, BA.2.75, and XBB spike sequences. We define the pandemic lineages based on the list of mutations described in outbreak.info.^54^ For each round of construct design, we built multiple sequence alignments for spike with both pre-pandemic sequences and pandemic spike sequences seen before the emergence of the variant used as the background for the design.

To generate the pre-pandemic alignment, we used 5 iterations of the jackhmmer iterative HMM-based homology search alignment tool,^52^ searching against the Uniref100 dataset^55^ and removed all sequences deposited after January 2020. To account for the growing number of pandemic sequences, we included unique pandemic sequences seen more than 100 times in the Global Initiative on Sharing All Influenza Data (GISAID) EpiCoV database (www.gisaid.org)^56^ at the time of emergence of the variant (Table S1). The resulting pandemic sequences and evolutionary alignments were combined and aligned with super5,^57^ version 5.1. We remove sequences that are aligned to less than 50% of the query sequence and remove residue positions that contain more than 70% gap characters. We down weighted redundant sequence clusters by assigning each protein sequence i a weight w(i)=1∕T, where T is the number of sequences in the alignment within a given hamming distance cutoff of t(0.01).

For the B.1 variant, two models were trained to assess the impact of incorporating SARS-CoV-2 sequences. The first model was trained exclusively on sequences available from pre-pandemic coronaviruses (n = 4,577 non-SARS-CoV-2 spike sequences; Table S1). For comparison purposes, the second model was updated to include SARS-CoV-2 spike sequences seen prior to May 2022 (n = 5,868, with 1,291 sequences from SARS-CoV-2 which represented ~9 independent sequences in model training due to high similarity) and used for a much smaller design round (6 of the 21 B.1 designs were using this model). For all other variants, model training included all SARS-CoV-2 spike sequences available up to the emergence of the respective variant (Tables S1). Due to the high similarity across the SARS-CoV-2 sequences, they at most represented 1.4% of the training dataset by effective number of weighted-redundant sequences.

#### EVE-Vax protein design algorithm

We use the EVEscape framework,^30^ which combines fitness, accessibility, and dissimilarity, to predict a mutant’s ability to escape antibody recognition. For the fitness (f) component we trained an EVE model,^50^ a Bayesian variational autoencoder (VAE) on the multiple sequence alignments. We estimate the relative fitness of each sequence as the log likelihood ratio between a mutated sequence and the wild type sequence. This ratio is itself approximated as the difference in Evidence Lower Bound (ELBO). We estimate ELBO values using twenty thousand Monte Carlo samples of the latent space. For the dissimilarity (d) component, we use both charge and hydrophobicity^58^ differences between each pair of amino acids and then assign each pair a dissimilarity value equal to the sum of the standard-scaled differences. For the accessibility (a) component, we selected spike structures from the RCSB Protein Data Bank (PDB) representative of both “open” and “closed” configurations (PDB IDs: 6VXX, 6VYB, 7CAB, and 7BNN). Using these structures, we calculated the weighted contact number (WCN) for each residue position i as $WCN_{i}=\sum_{j\neq l}(r_{ij}^{-2})$ where $r_{ij}$ is the distance between the geometric centers of the side chain of the residues occupying sites i and j. Each position is then assigned the minimum WCN across all structures. We use the negative WCN as a measure of residue accessibility to antibodies.

For each mutation, we combined the fitness, accessibility, and dissimilarity scores using a temperature scaled logistic function to get a single escape score for each individual mutation. The score of a mutation m is calculated as

$$EVEscape(m)=logistic\left(T_{t}^{-1}*F_{t}^{m}\right)*logistic\left(T_{a}^{-1}*F_{a}^{m}\right)*logistic\left(T_{d}^{-1}*F_{d}^{m}\right)$$

where $T_{i}$ is the temperature scaling and $F_{i}$ is the standardized vector for factor i. We then take the log transform of the product. We modified the temperature parameters compared to the values used previously^30^ by increasing the contribution of the fitness component, $T_{f}=1$, $T_{a}=8$, and $T_{d}=16$. The modular design of this framework enhances model interpretability by allowing us to assess the relative contributions of each of the three components to a mutation’s overall escape score (Figure S1).

We designed 83 constructs on the backgrounds of five different spike VOC sequences: B.1 (ancestral strain with D614G), BA.4/5, BA.2.12.1, BA.2.75, and XBB. For each background variant we first scored all possible single amino acid substitutions to spike. Note that we consider amino acid mutations involving any number of nucleotide changes rather than focusing on only single nucleotide changes which are by far the most common (more than 99%) mutations seen in GISAID.^56^ To design constructs, we considered single amino acid mutations in the top 1% of EVEscape score. We then generated and scored all possible double mutants. Lastly, we combined highly fit double mutants and scored them to create higher order constructs.

#### Recombinant DNA sequence plasmid design

All recombinant DNA work was conducted according to protocols approved by the University of Massachusetts Chan Medical School Institutional Biosafety Committee. We designed a pDMJ2 plasmid where spike expression is under the control of a cytomegalovirus immediate early promoter (CMV-IE) fused to the human T lymphotropic virus type 1 (HTLV-1) 5′ UTR.^59^ Transcription termination is directed by a bovine growth hormone polyadenylation signal (bGH-PolyA). The pDMJ2 plasmid carries an origin of replication (ori) from ColE1 and an Ampicillin resistance gene. spike sequences were inserted between the *XbaI* and *BamHI* restriction sites (Table S2; Figure S1).

An ancestral B.1 spike sequence was codon optimized by Twist Biosciences with a deletion of the terminal 18 amino acids to remove the endoplasmic reticulum retention signal.^60^ This sequence was used as the base sequence on which other constructs were designed to minimize effects of nucleotide sequence on protein synthesis efficiency. Specifically, to generate spike sequences of VOCs and designed constructs, we mutated individual codon positions to the most commonly used codon in human cells encoding the mutant amino acid. We repeated this for all mutations in a given sequence. If a restriction site was introduced, the second most common codon was used.

#### Infectivity assays with SARS-CoV-2 spike-pseudotyped lentiviral particles

All infectivity assays utilized replication-incompetent lentiviral pseudotypes and were conducted according to protocols approved by the University of Massachusetts Chan Medical School Institutional Biosafety Committee. HIV-1-derived virions bearing a luciferase reporter gene, and pseudotyped with a SARS-CoV-2 spike variant, were produced by transfection of HEK293T cells, as previously reported.^34^ Each S protein expression plasmid was separately transfected into HEK293 cells with plasmids encoding HIV-1 structural proteins and enzymes. Separate plasmids were transfected that encode RNAs with HIV-1 *cis*-acting signals for packaging and replication and either GFP or luciferase (Luc) reporter cassettes. Fresh media was added after spinfection and cells were incubated prior to analysis. The supernatant from the transfected cells was collected 72 hours post-transfection and filtered (0.45 μM, Avantor^™^) to remove cellular debris. For each condition tested, multiple virus stocks were produced, and each stock was tested in triplicate after vector particle normalization by using reverse-transcriptase activity. Virion yield in each transfection supernatant was normalized using our in-house exogenous reverse transcriptase (RT) activity assay.^34,61^ As in Yurkovetskiy et al.,^34^ 5 μL transfection supernatant was mixed with 5 μL 0.25% Triton X-100, 50 mM KCl, 100 mM Tris-HCl pH 7.4, and 0.4 U/μL RiboLock RNase inhibitor, and then diluted 1:100 in 5 mM (NH_4_)_2_SO_4_, 20 mM KCl, and 20 mM Tris-HCl pH 8.3. 10 μL of this was then added to a single-step, RT-PCR assay with 35 nM MS2 RNA (IDT) as template, 500 nM of each primer (5′-TCCTGCTCAACTTCCTGTCGAG-3′ and 5′-CACAGGTCAAACCTCCTAGGAATG-3′), and 0.1 μL hot-start Taq DNA polymerase (Promega, Madison, WI) in 20 mM Tris-Cl pH 8.3, 5 mM (NH_4_)_2_SO_4_, 20 mM KCl, 5 mM MgCl_2_, 0.1 mg/mL BSA, 1/20,000 SYBR Green I (Invitrogen), and 200 μM dNTPs in total 20 μL reaction. The RT-PCR reaction was carried out in a Biorad CFX96 real-time PCR detection system with the following parameters: 42° C for 20 minutes, 95° C for 2 minutes, and 40 cycles [95° C for 5 seconds, 60° C for 5 seconds, 72° C for 15 seconds, and acquisition at 80° C for 5 seconds].

Supernatant containing pseudovirions was then added to HEK293T cells that were previously transduced with puromycin- and blasticidin-resistant lentivectors to stably express the human angiotensin converting enzyme 2 (ACE2) and the transmembrane serine protease 2 (TMPRSS2),^34^ respectively (HEK293T-ACE2-TMPRSS2). 16 hours prior to transduction, HEK293T-ACE2-TMPRSS2 cells were seeded in 96 well plates. HEK293T cells were plated at 7.5 × 10^3^ cells per well. Cells were incubated in virus-containing media for 16 hours at 37° C when fresh medium was added to cells and returned to incubation for another 56 hours.. Luciferase activity on target cells was measured as an indication of transduction efficiency, using the Steady-Glo^®^ Luciferase Assay System (Promega Corporation) read on a Promega GloMax Discover machine. All plasmids were deposited to Addgene (Table S2). All experiments were performed in triplicate. Sequences were considered non-functional if they had the same measured transduction (Relative Light Units; RLU) as the negative control (pDMJ2 without spike inserted, <1 x 10^4^ RLU).

#### Neutralization assays with SARS-CoV-2 spike-pseudotyped lentiviral particles

Neutralizing activity against SARS-CoV-2 pseudovirus was measured using a single-round infection assay in 293T/ACE2 target cells. Pseudotyped virus particles were produced in 293T/17 cells (ATCC) by co-transfection of pDMJ2 plasmids encoding codon-optimized SARS-CoV-2 spike variant, packaging plasmid pCMV R8.2, and luciferase reporter plasmid pHR’ CMV-Luc. Packaging and luciferase plasmids were kindly provided by Dr. Barney Graham (NIH, Vaccine Research Center). The 293T cell line stably overexpressing the human ACE2 cell surface receptor protein was kindly provided by Drs. Michael Farzan and Huihui Ma (The Scripps Research Institute). For neutralization assays, serial dilutions of patient serum samples were performed in duplicate followed by addition of pseudovirus. Pooled serum samples from convalescent COVID-19 patients or pre-pandemic normal healthy serum (NHS) were used as positive and negative controls, respectively. Plates were incubated for 1 hour at 37 C followed by addition of 293/ACE2 target cells (1x10^4^ /well). Wells containing cells and pseudovirus (without sample) or cells alone acted as positive and negative infection controls, respectively. Assays were harvested on day 3 using Promega BrightGlo luciferase reagent and luminescence detected with a Promega GloMax luminometer. Titers are reported as the dilution of serum that inhibited 50% or 80% virus infection (ID_50_ and ID_80_ titers, respectively).

#### Analysis of proportion of viable sequences at different mutational depths

We used the raw data from four deep mutation scans (viral and non-viral) to quantify the likelihood of obtaining functional sequences with multiple mutations compared to a wild type sequence. We analyzed the SARS-CoV-2 RBD data from Starr et al.,^31^ the full spike SARS-CoV-2 data from Dadonaite et al.,^29^ the Lassa glycoprotein complex (GPC) data from Carr et al.,^32^ and the *Clytia gregaria* green fluorescent protein (GFP) data from Somermeyer et al.^33^

In the RBD dataset, mutations were introduced using error-prone PCR resulting in 135,386 unique mutant RBDs with up to 11 amino acid mutations. The expression of each sequence was measured as the change in mean fluorescence intensity, Δlog(MFI), relative to the unmutated SARS-CoV-2 RBD. We used sequences containing synonymous mutations (labeled viable) and sequences with premature stop codons (labeled nonviable) to train a logistic regression for classifying missense variants as either viable or nonviable. The fitted model had an intercept of 4.66 and a coefficient of 1.39. The decision threshold estimated for expression scores was −1.56 Δlog(MFI) with variants having expression values less than this boundary classified as non-viable, and variants with expression scores greater than the boundary classified as viable. When analysing this same data, Greaney et al.^15^ used an expression threshold of −1 Δlog(MFI) to classify variants as nonviable since that was the expression deficit for mutations to core disulfide residues. More recently, Greaney et al.^13^ used an expression threshold of −1.5 Δlog(MFI). Our estimated decision threshold of −1.56 Δlog(MFI) is therefore in-line with previous biologically-informed cutoffs.

We repeated the logistic regression analysis described above on the other three datasets. In the full spike dataset from Dadonaite et al.,^29^ only mutations seen in the pandemic were introduced to the full spike Delta variant in higher-order combinations. The dataset included 191,418 unique variants containing up to 38 mutations. For each variant, a functional score was estimated based on its relative frequency in the spike- versus VSV-G-pseudotyped libraries. The fitted model had an intercept of 6.11 and a coefficient of 3.91. The decision threshold was estimated to be a functional score of −3.34, with variants having values lower than this cutoff classified as non-viable and variants with higher values classified as viable.

In the Lassa GPC data from Carr et al.,^32^ the libraries were created using error-prone PCR. The dataset included 66,793 unique variants containing up to 34 mutations. For each variant, a functional score was estimated based on cell entry measurements in 293T cells. The fitted model had an intercept of 2.5 and a coefficient of 0.8. The decision threshold was estimated to be a functional score of −3.15, with variants having values lower than this cutoff classified as non-viable and variants with higher values classified as viable.

In the *Clytia gregaria* GFP dataset from Somermeyer et al.,^33^ the libraries were created using error-prone PCR. The dataset included 26,165 unique variants containing up to 230 mutations. For each variant, a functional score was estimated based on the log of the green fluorescence intensity. The fitted model had an intercept of −46.6 and a coefficient of 10.9. The decision threshold was estimated to be a functional score of 4.25, with variants having values lower than this cutoff classified as non-viable and variants with higher values classified as viable.

#### Analysis of escape mutation frequencies in the public database

We analyzed experimental data from 20 high-throughput deep mutational scanning studies (Table S5) that measured escape mutations against monoclonal antibodies (mAbs) or sera.^2,3,6,13-25,27-29,62^ For each study, mutations were assigned an escape score, calculated as either the maximum or mean score across conditions (antibodies or sera). Escape mutations were defined as nonsynonymous mutations within one nucleotide of the wildtype amino acid and ranked in the top 5% of escape scores for each study. The proportion of escape mutations observed more than 1,000 times throughout the pandemic (using GISAID^56^ downloaded on Dec 19 2024) is reported. Proportions for each study independently and with different thresholds (top 1%, 5% and 10%) are shown in Figure S4.

To estimate the cumulative proportion of escape mutations across studies (Figure 5A), escape mutations were initially defined based on the earliest available experimental data. When subsequent studies became available, we updated the set of escape mutations to include any additional mutations identified by the most recent study which were not in the prior set of escape mutations. This iterative approach accounts for variability between studies conducted in different laboratories, at different times, and under differing experimental conditions, while maintaining the utility of earlier studies performed on previous variants of concern. The same protocol was applied to define escape mutations predicted by computational models.

#### Comparison to assay-derived constructs designed using high throughput experiments

We compare the EVE-Vax designed constructs to multi-mutant constructs designed from large-scale experimental studies of escape from polyclonal sera or thousands of monoclonal antibodies.^11,22,63^ For B.1 designs, we compare against designs from Schmidt et al.,^11^ with matched sera: High Titer Convalescent Wildtype (ours: High convalescent WT titer pool (n=5), theirs: RU27 (n=27)), Convalescent Wildtype (ours: Convalescent WT (n=15), theirs: Ran21, (n=21)), and Vaccine (ours: Primary Vaccination (n=25), theirs: Vac14 (n =14); Figure S5). Their designs included mutations chosen from passaging a (rVSV)/SARS-CoV-2 chimeric virus in the presence of plasmas for up to six passages, and mutations were chosen based on their effects on plasma neutralization sensitivity and distribution throughout the spike protein. The constructs with the highest average fold reduction were B.1-8a (ours) and PSM1-1 (theirs).

For BA.5, we compare against the 8 BA.5 RBD designs generated by Cao et al.,^22^ with matched sera: Boost (ours: Boost (n=25), theirs: CoronaVac 3 doses (n= 40)), BA.1 BTI (ours: BA.1 BTI (n=15), theirs: BA.1 BTI post 3 doses of CoronaVac, (n= 50)), BA.2.12.1 BTI (ours: BA.2.12.1 BTI (n=5), theirs: BA.2 BTI post 3 doses of CoronaVac (n=50)) and BA.5 BTI (ours: BA.5 BTI (n=5), theirs: BA.5 BTI post 3 doses of CoronaVac, (n=36); Figure S5). Their designs included mutations chosen from measuring high-throughput scans of human-ACE2 binding affinity, RBD expression and neutralizing activity across 3,051 BA.2-neutralizing antibodies from varied immune backgrounds, as well as codon constraints on each residue. The constructs with the highest average fold reduction were BA.5-5a (ours) and BA.5-S8 (theirs).

For BA.2.75, we compare against the 8 BA.2.75 RBD designs generate by Cao et al.,^22^ with matched sera: Boost (ours: Boost (n=25), theirs: CoronaVac 3 doses (n= 40)), BA.1 BTI (ours: BA.1 BTI (n=15), theirs: BA.1 BTI post 3 doses of CoronaVac, (n= 50)), BA.2.12.1 BTI (ours: BA.2.12.1 BTI (n=5), theirs: BA.2 BTI post 3 doses of CoronaVac (n=50)) and BA.5 BTI (ours: BA.5 BTI (n=5), theirs: BA.5 BTI post 3 doses of CoronaVac, (n=36); Figure S5). Their designs included mutations chosen from measuring high-throughput scans of human-ACE2 binding affinity, RBD expression and neutralizing activity across 3,051 BA.2-neutralizing antibodies from varied immune backgrounds, as well as codon constraints on each residue. The constructs with the highest average fold reduction were BA.2.75-4c (ours) and BA.2.75-S7 (theirs).

For XBB, we compare against the 7 XBB.1.5 RBD designs generated by Yisimayi et al.,^63^ with matched sera: BA.1 BTI (ours: BA.1 BTI (ours: BA.1 BTI (n=15), theirs: BA.1 BTI prior to BA.5/BF.7 reinfection (n=26)), BA.2.12.1 BTI (ours: BA.2.12.1 BTI (n=5), theirs: BA.2 BTI prior to BA.5/BF.7 reinfection, (n=19)), and BA.5 BTI (ours: BA.5 BTI (n=5), theirs: Reinfection with BA.5 or BF.7 after BA.1 or BA.2 infection without vaccination (n=12); Figure S5). Their designs included mutations chosen from measuring high-throughput scans of human-ACE2 binding affinity, RBD expression and neutralizing activity across 1,816 SARS-CoV-2 RBD-targeting antibodies from varied immune backgrounds, as well as codon constraints on each residue. The constructs with the highest average fold reduction were XBB-9a relative to XBB (ours) and XBB.1.5-S5 relative to XBB.1.5 (theirs).

### QUANTIFICATION AND STATISTICAL ANALYSIS

We use the Wilcoxon signed rank test from SciPy for comparing differences in neutralization, with the p-value indicated and fold reduction in geometric mean ID_50_ titers shown. For XBB designs the Delta convalescent and primary vaccine sera pool were not included since the serum pools were depleted at the time these constructs were made. The statistical test in this case only considered the remaining sera.

For antigenic cartography, we use Racmacs^51^ to perform multidimensional scaling (MDS) on our titration data measuring the neutralization sensitivity of all VOCs and designs for each serum pool. MDS is used to create a 2-dimensional representation of the pairwise antigenic distance between each VOC or design, as dictated by how each is neutralized by the different sera pools. MDS pairwise distances are consistent, which can be seen across pairs of variant-variant, variant-design, and design-design (Figure S3). This allows us to visualize the trajectory of evolution in antigenic space.

## Supplementary Material

1-s2.0-S1074761325001785-mmc1

1-s2.0-S1074761325001785-mmc2

1-s2.0-S1074761325001785-mmc3

1-s2.0-S1074761325001785-mmc4

1-s2.0-S1074761325001785-mmc5

1-s2.0-S1074761325001785-mmc6

Supplemental information can be found online at https://doi.org/10.1016/j.immuni.2025.04.015.

## Figures and Tables

**Table:** KEY RESOURCES TABLE

REAGENT or RESOURCE	SOURCE	IDENTIFIER
Antibodies		
Human polyclonal sera - B.1 convalescent infection	Serum blood donation. This paper.	N/A
Human polyclonal sera - Delta convalescent infection	Serum blood donation. This paper.	N/A
Human polyclonal sera - B.1 (Moderna) Primary Vaccination	Serum blood donation. This paper.	N/A
Human polyclonal sera - B.1 (Pfizer) Primary Vaccination	Serum blood donation. This paper.	N/A
Human polyclonal sera - B.1 (Moderna) Boost	Serum blood donation. This paper.	N/A
Human polyclonal sera - B.1 (Pfizer) Boost	Serum blood donation. This paper.	N/A
Human polyclonal sera - B.1 & BA.4/5 Bivalent Boost	Serum blood donation. This paper.	N/A
Human polyclonal sera - Delta Vaccine Breakthrough Infection	Collected from patients^47-49^	N/A
Human polyclonal sera - BA.1 Vaccine Breakthrough Infection	Collected from patients^47-49^	N/A
Human polyclonal sera - BA.2.12.1 Vaccine Breakthrough Infection	Collected from patients^47-49^	N/A
Human polyclonal sera - BA.4/5 Vaccine Breakthrough Infection	Collected from patients^47-49^	N/A
NHP polyclonal sera - bivalent mRNA Vaccine Boost	Collected from NHP (Cohen et al.^38^)	N/A
NHP polyclonal sera - mosaic-8b nanoparticle Boost	Collected from NHP (Cohen et al.^38^)	N/A
NHP polyclonal sera - homotypic nanoparticle Boost	Collected from NHP (Cohen et al.^38^)	N/A
Bacterial and virus strains		
See Table S2 for 104 viral strains used	This paper	See Table S2 for addgene identifiers
Chemicals, peptides, and recombinant proteins		
Filters, 0.45 μM	Avantor	Cat#76479-020
Critical commercial assays		
RT Assay	Yurkovetskiy et al.^34^	N/A
Steady-Glo Luciferase Assay System	Promega	Cat#E2550
Bright-Glo Luciferase Assay System	Promega	Cat#E2650
HIV pseudotyping assay	Yurkovetskiy et al.^34^	N/A
Deposited data		
SARS-CoV-2 Sequences	GISAID^49^	N/A
Spike structures in open and closed conformations	PDB	PDB:6VXX; PDB:6VYB; PDB:7CAB; PDB:7BNN
Raw and analyzed data	This paper	Tables S3 and S4 and https://github.com/debbiemarkslab/Vax_design
Experimental models: Cell lines		
HEK 293T cells with stably overexpressed ACE2	Drs. Michael Farzan and Huihui Ma (The Scripps Research Institute)	N/A
HEK 293T/17 cells	ATCC	Cat#CRL-11268
HEK293T cells with stably expressed ACE2/TMPRSS2	Yurkovetskiy et al.^34^	N/A
HEK293T cells	ATCC	Cat#CRL-3216
Recombinant DNA		
Plasmid: pDMJ2	This paper	N/A
Plasmid: pCMV-R8.2	Dr. Barney Graham (NIH Vaccine Research Center)	N/A
Plasmid: pHR’CMV-Luc	Dr. Barney Graham (NIH Vaccine Research Center)	N/A
Software and algorithms		
EVE-Vax	This paper	https://github.com/debbiemarkslab/Vax_design
EVEscape	Thadani et al.^30^	https://github.com/OATML-Markslab/EVEscape
EVE	Frazer et al.^50^	https://github.com/OATML-Markslab/EVE
Racmacs	Wilks^51^	https://acorg.github.io/Racmacs/
Jackhmmer	Johnson et al.^52^	http://hmmer.org/download.html

## Highlights

- Developed EVE-Vax, a computational method for designing antigens
- Designed and tested 83 poly-mutant SARS-CoV-2 spike proteins
- EVE-Vax designs foreshadowed immune escape observed in natural pandemic variants
- EVE-Vax designs offer an approach for proactive vaccine and therapeutic evaluation
