# Supplementary material for: Computationally designed proteins mimic antibody immune evasion in viral evolution
Source: Immunity. Author manuscript; Available in PMC 2026 Apr 25. (PMC13110123; doi:10.1016/j.immuni.2025.04.015)
Supplement: 1-s2.0-S1074761325001785-mmc1 [file NIHMS2161490-supplement-1-s2_0-S1074761325001785-mmc1.pdf]

Immunity, Volume *58*

Supplemental information

Computationally designed proteins mimic antibody immune evasion in viral evolution

Noor Youssef, Sarah Gurev, Fadi Ghantous, Kelly P. Brock, Javier A. Jaimes, Nicole N. Thadani, Ann Dauphin, Amy C. Sherman, Leonid Yurkovetskiy, Daria Soto, Ralph Estanboulieh, Ben Kotzen, Pascal Notin, Aaron W. Kollasch, Alexander A. Cohen, Sandra E. Dross, Jesse Erasmus, Deborah H. Fuller, Pamela J. Bjorkman, Jacob E. Lemieux, Jeremy Luban, Michael S. Seaman, and Debora S. Marks

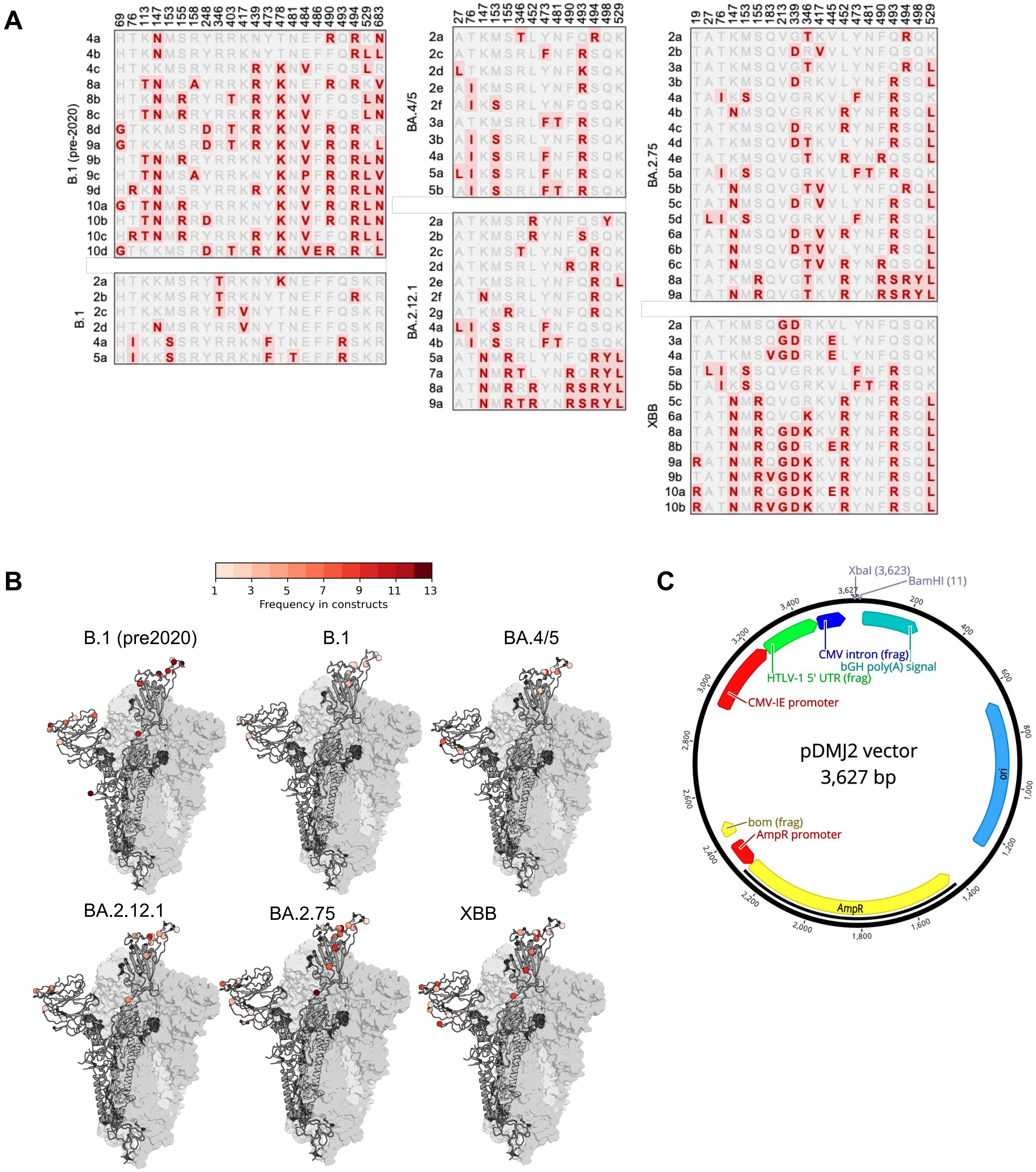

A
B.1 (pre-2020)
B.1
BA.4/5
BA.2.12.1
BA.2.75
XBB
B
Frequency in constructs
1 3 5 7 9 11 13
B.1 (pre2020)
B.1
BA.4/5
BA.2.12.1
BA.2.75
XBB
C
pDMJ2 vector
3,627 bp
XbaI (3,623)
BamHI (11)
CMV intron (frag)
bGH poly(A) signal
HTLV-1 5' UTR (frag)
CMV-IE promoter
ori
bom (frag)
AmpR promoter
AmpR

D

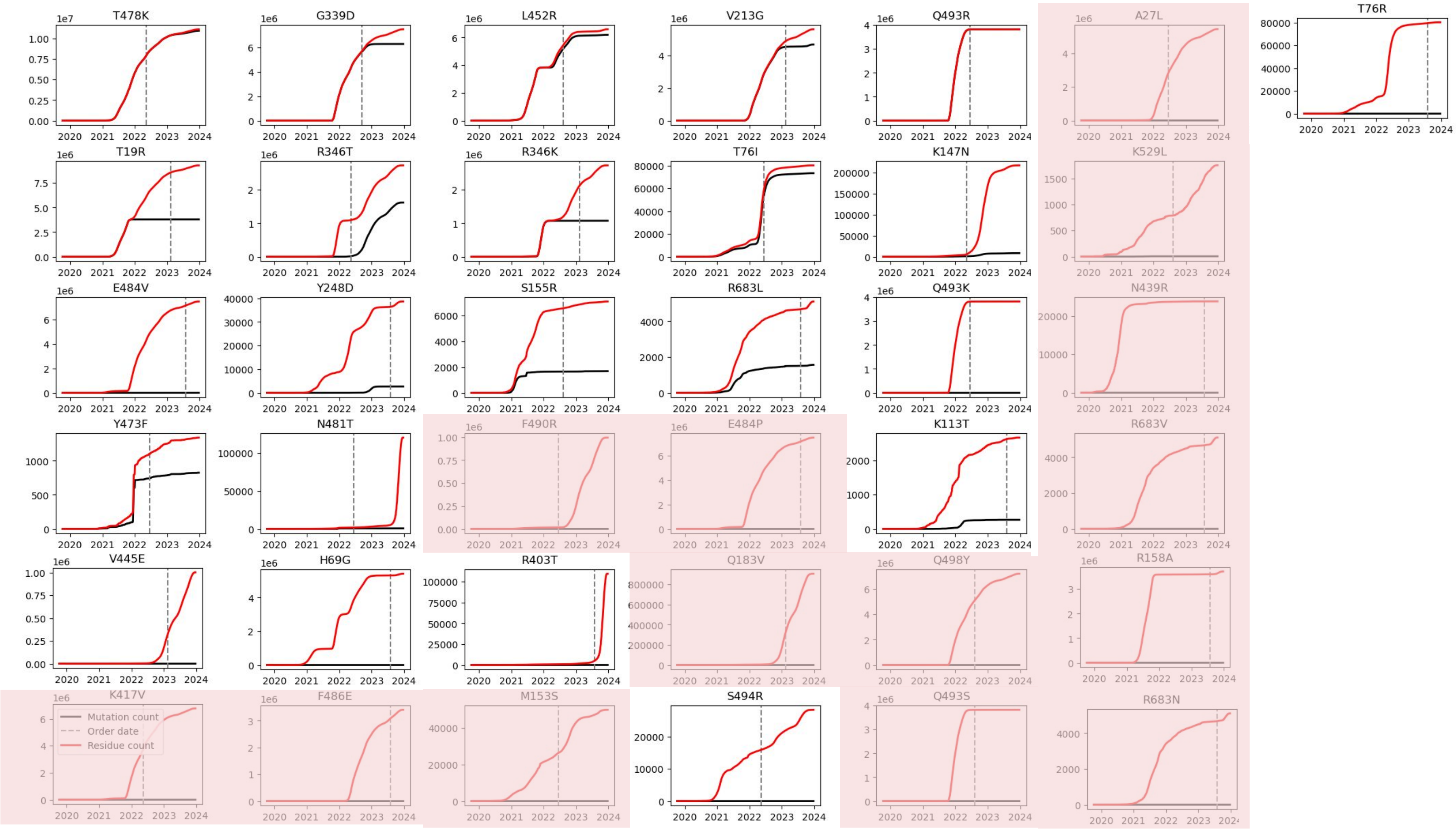

E

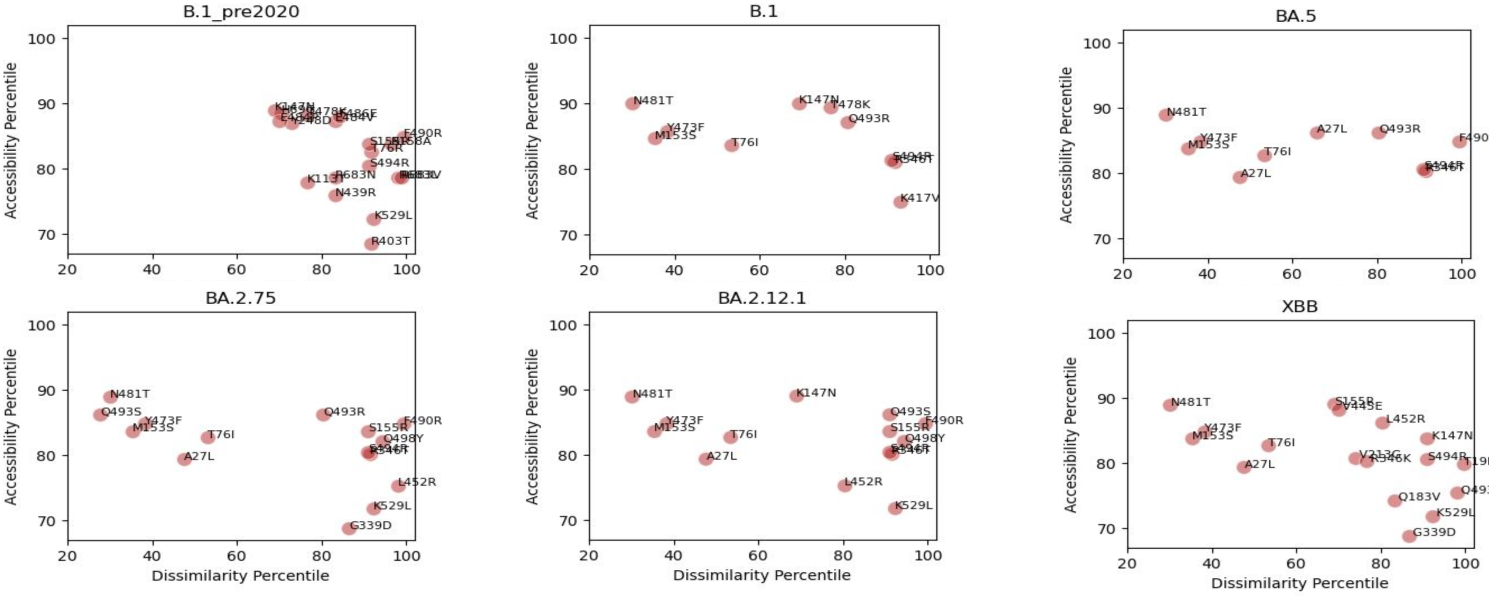

Figure S1: Overview of designed sequences. Related to Figure 1. **(A)** Designed Spikes with two or more mutations (**B**) Designed mutations on all background VoCs (PDB ID: 7BNN). Coloring indicates frequency with which a given residue was mutated. Mutations in variant background sequences marked with black spheres. **(C)** pDMJ2 plasmid map (3,627 bp) CMV-IE promoter: Cytomegalovirus immediate early promoter; HTLV-1 5' UTR (frag): human T lymphotropic virus type 1 (HTLV-1) 5' UTR fragment, CMV intron (frag): Cytomegalovirus intron fragment; bGH poly(A) signal: bovine growth hormone polyadenylation signal; ori: ColE1 origin of replication; AmpR: Ampicillin resistance gene; AmpR promoter: Ampicillin resistance promoter; bom (frag): ColE1 basis of mobility fragment. *XbaI* and *BamHI* restriction sites. (**D**) Count of designed mutation throughout the COVID19 pandemic. Black line: cumulative mutation count in GISAID [S1]. Red line: cumulative count of any mutations at that residue. Gray dashed line: date construct containing that particular mutation was ordered. Designed mutations requiring more than one nucleotide change (pink background), were rare in the pandemic. (**E**) Accessibility and dissimilarity components of designed mutations**.**

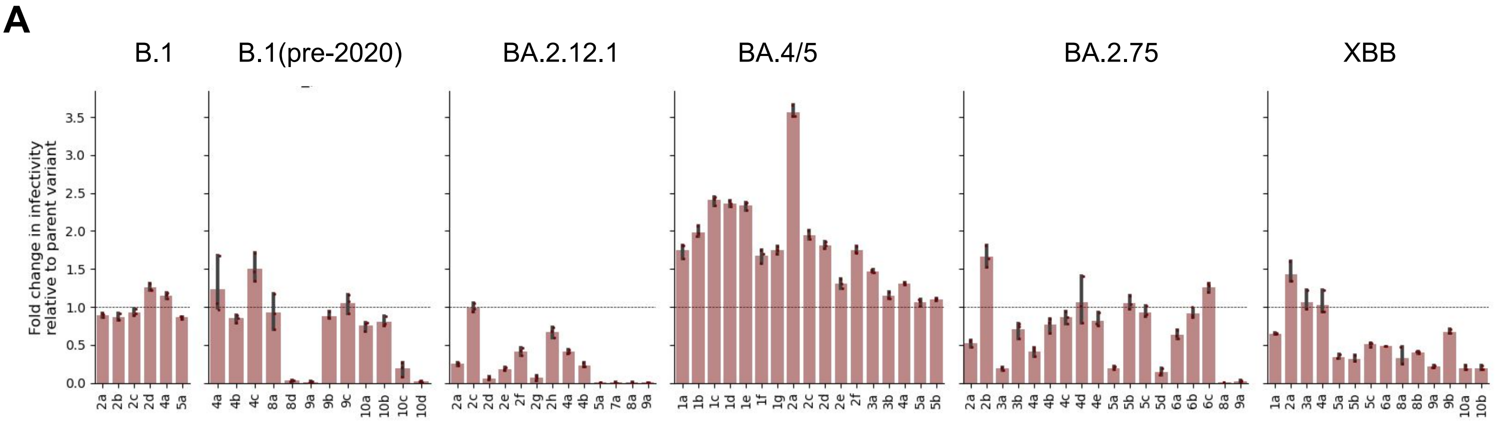

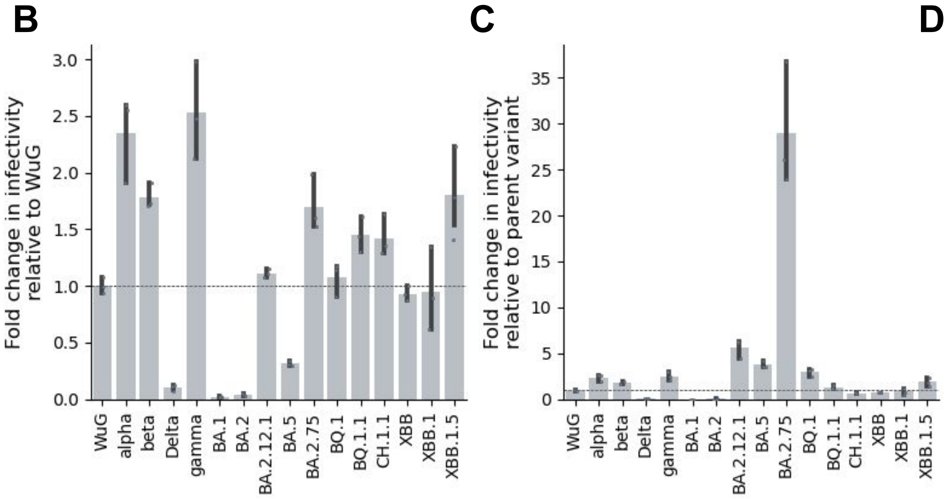

D

Variant	Parent Variant
B.1-D614<br>Alpha<br>Beta<br>Delta<br>Gamma<br>BA.1<br>BA.2	B.1
BA.2.12.1<br>BA.4/5<br>BA.2.75	BA.2
BQ.1	BA.4/5
BQ.1.1	BQ.1
XBB<br>CH.1.1	BA.2.75
XBB.1	XBB
XBB.1.5	XBB.1

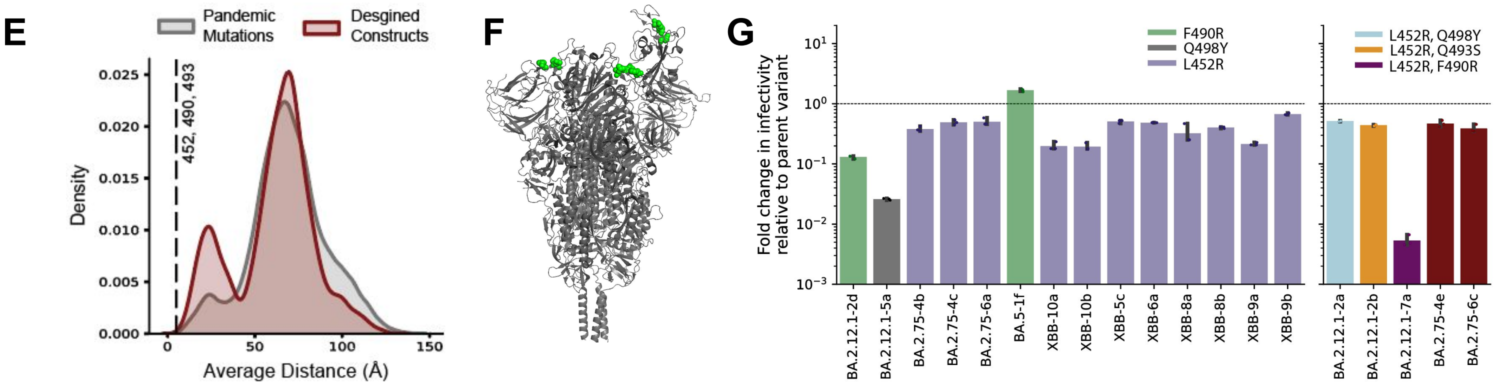

Figure S2: Infectivity Data. Related to Figure 2. **(A)** Fold change in infectivity of designed constructs relative to the base variants of concern (VOC). Error bars are 95% confidence intervals. **(B)** Fold change in infectivity of VOCs relative to B.1 (WuG). **(C)** Fold change in infectivity of VOCs relative to their parent variant. **(D)** Summary of parent variants for VOCs. (**E**) Four designed constructs which were not infectious shared a unique subset of mutations: L452R, F490R, Q493S, and Q498Y. The three residues 452, 490, and 493 are closer in average distance than the average distance of any triplet of mutations in any pandemic strains or in any other designed constructs. **(F)** Three proximal residues (452, 490, 493) are shown on Spike structure (PDB ID: 7BNN). **(G)** Constructs containing some of these mutations individually, or in pairs, remain infectious. Error bars are 95% confidence intervals.

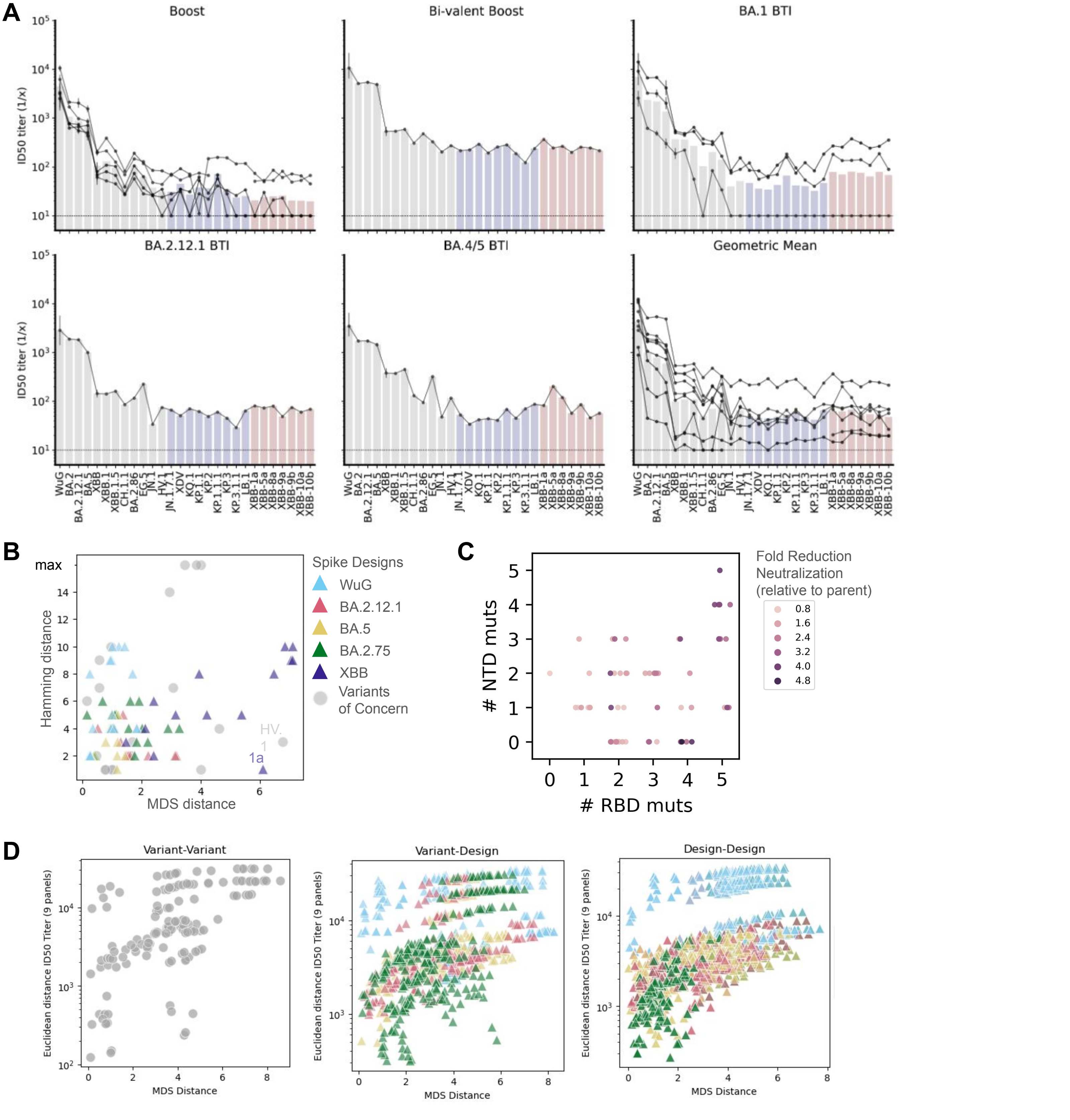

Figure S3: Designed constructs foreshadow antibody evasion of later variants. Related to Figure 3. **(A)** Designed constructs on XBB have similar levels of neutralization susceptibility as recently emerging variants (KP*). Neutralizing ID50 titers across sera panels from individuals that were boosted (Boost), bi-valent boosted (Bi-valent boost), or those with breakthrough infections (BTI) with BA.1, BA.2.12.1, and BA.4/5 variants. Each point is a pool of 5 individual serum samples and bars represent the geometric mean across pools. In the last subplot, each point is the geometric mean given a specific sera panel, and bars are the geometric means across all panels. Red: XBB designed constructs. Blue: most recent variants of concern. Grey: previously circulating variants of concern. **(B)** Antigenic distances are consistent and not entirely dependent on hamming distance. Antigenic distance (MDS, Fig 3A) versus hamming distance of Spike sequences from their parent variant. Some designs or variants have outsized impacts on antigenic distance relative to their number of mutations (e.g., pandemic variant HV.1 and design XBB-1a). Designs are colored by their parent variant. The maximum hamming distance shown is 16. **(C)** Designs broken down by their number of NTD vs. RBD mutations, colored by their fold reduction in neutralization relative to their parent variant. (**D**) MDS distances are consistent with euclidean distances in ID50 titers across the 9 sera panels, when looking between all tested variant-variant, variant-design, and design-design pairs.

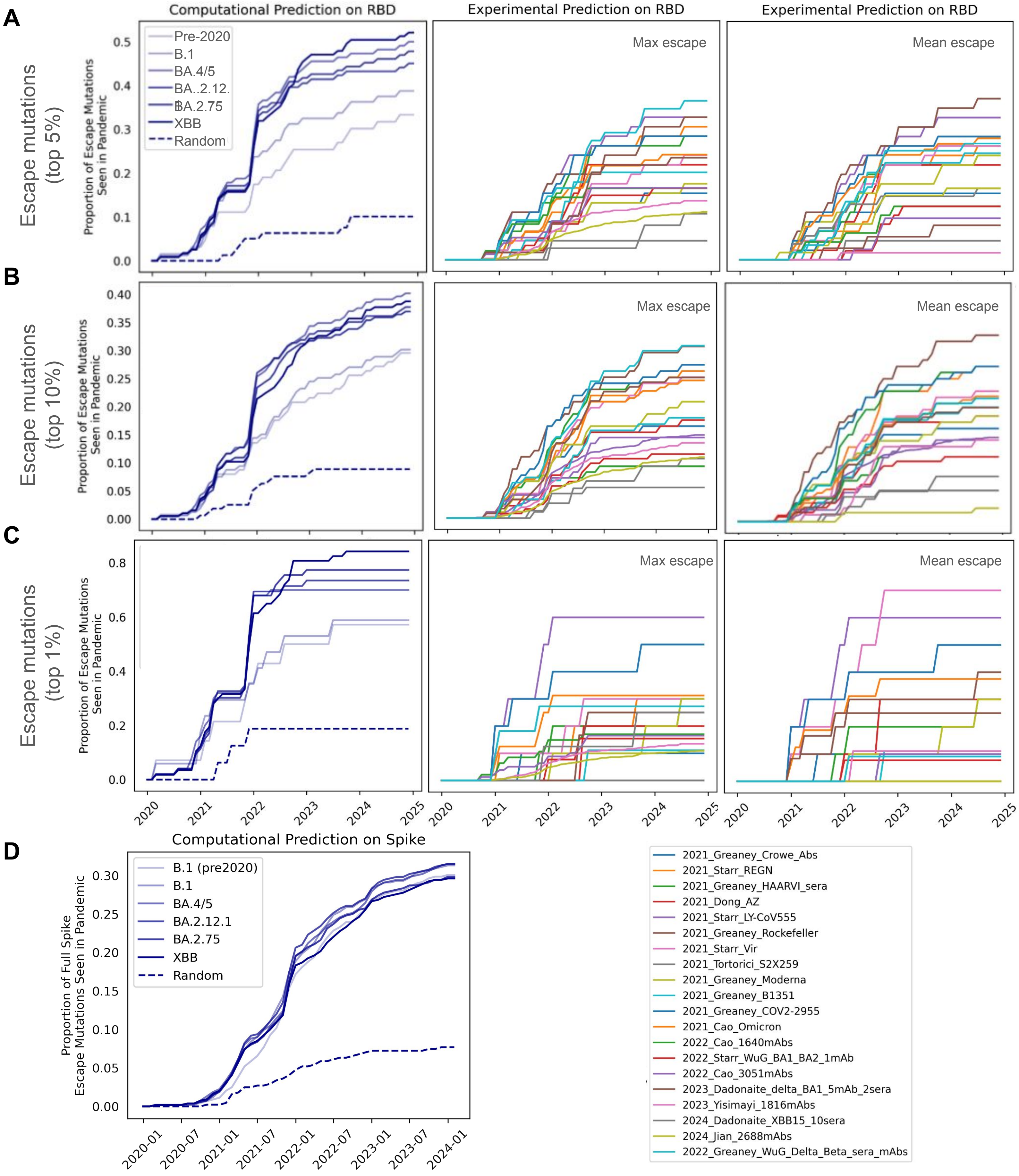

Figure S4: EVEscape better predicts later pandemic mutations than high throughput experiments and has improved performance when including pandemic sequences in training. Related to Figure 5. **(A,B,C)** Proportions of escape mutations that have been seen more than 1000 times in GISAID as predicted by EVEscape (with and without SARS-CoV-2 sequences) across the RBD (left), mutational scans where escape mutations for each study is defined as the maximum (center) or mean (right) escape across tested mAbs or sera. Escape mutations are defined as the top 5% (**A**), 10% (**B**) or 1% (**C**). (**D**) Proportion of computational predicted across the full Spike protein**.**

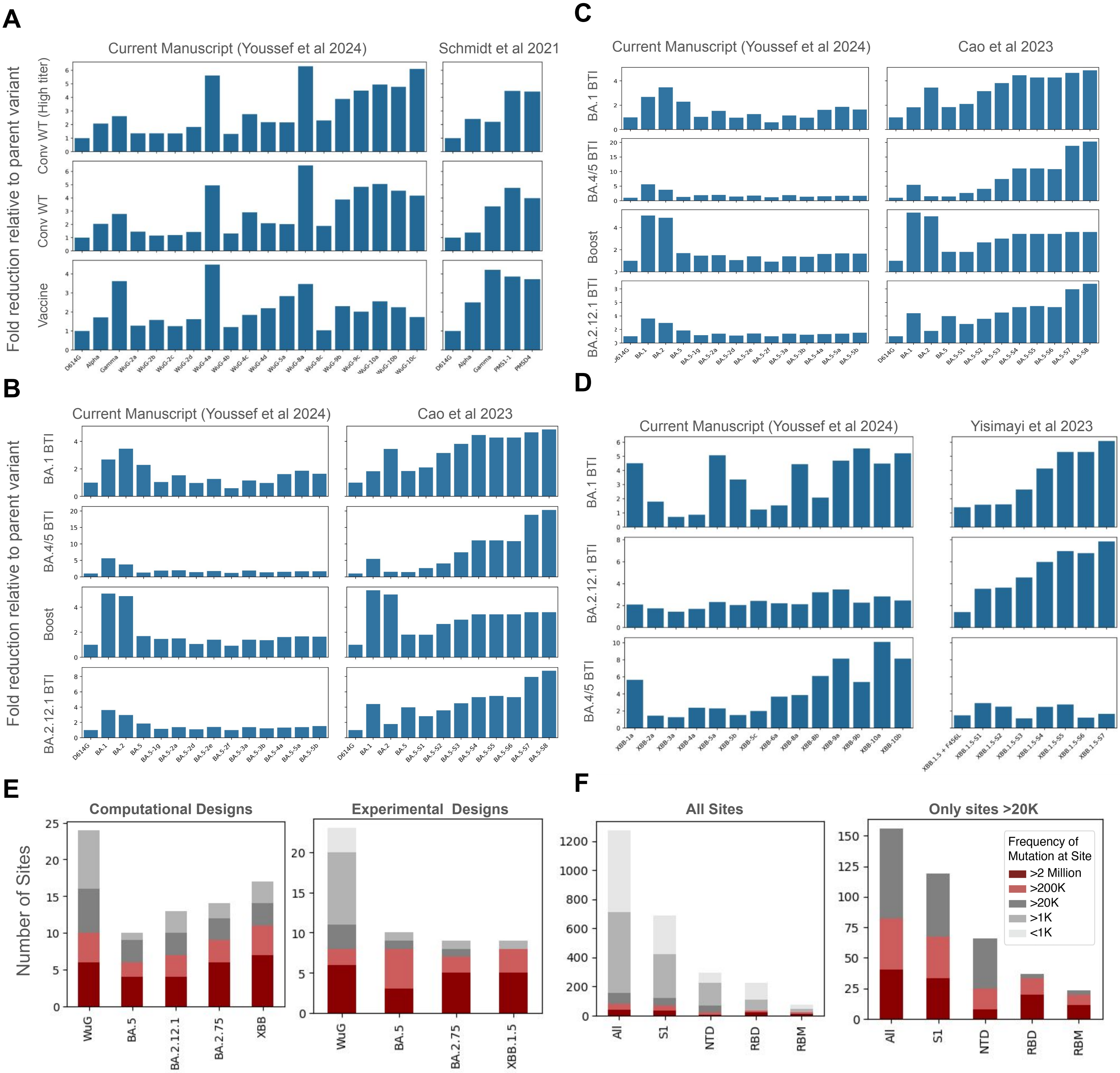

A
Current Manuscript (Youssef et al 2024)
Schmidt et al 2021
Fold reduction relative to parent variant
Conv WT (High titer)
Conv WT
Vaccine
B
Current Manuscript (Youssef et al 2024)
Cao et al 2023
BA.1 BTI
BA.4/5 BTI
Boost
BA.2.12.1 BTI
C
Current Manuscript (Youssef et al 2024)
Cao et al 2023
D
Current Manuscript (Youssef et al 2024)
Yisimayi et al 2023
E
Computational Designs
Experimental Designs
Number of Sites
WuG
BA.5
BA.2.12.1
BA.2.75
XBB
XBB.1.5
F
All Sites
Only sites >20K
Frequency of Mutation at Site
>2 Million
>200K
>20K
>1K
<1K
All
S1
NTD
RBD
RBM

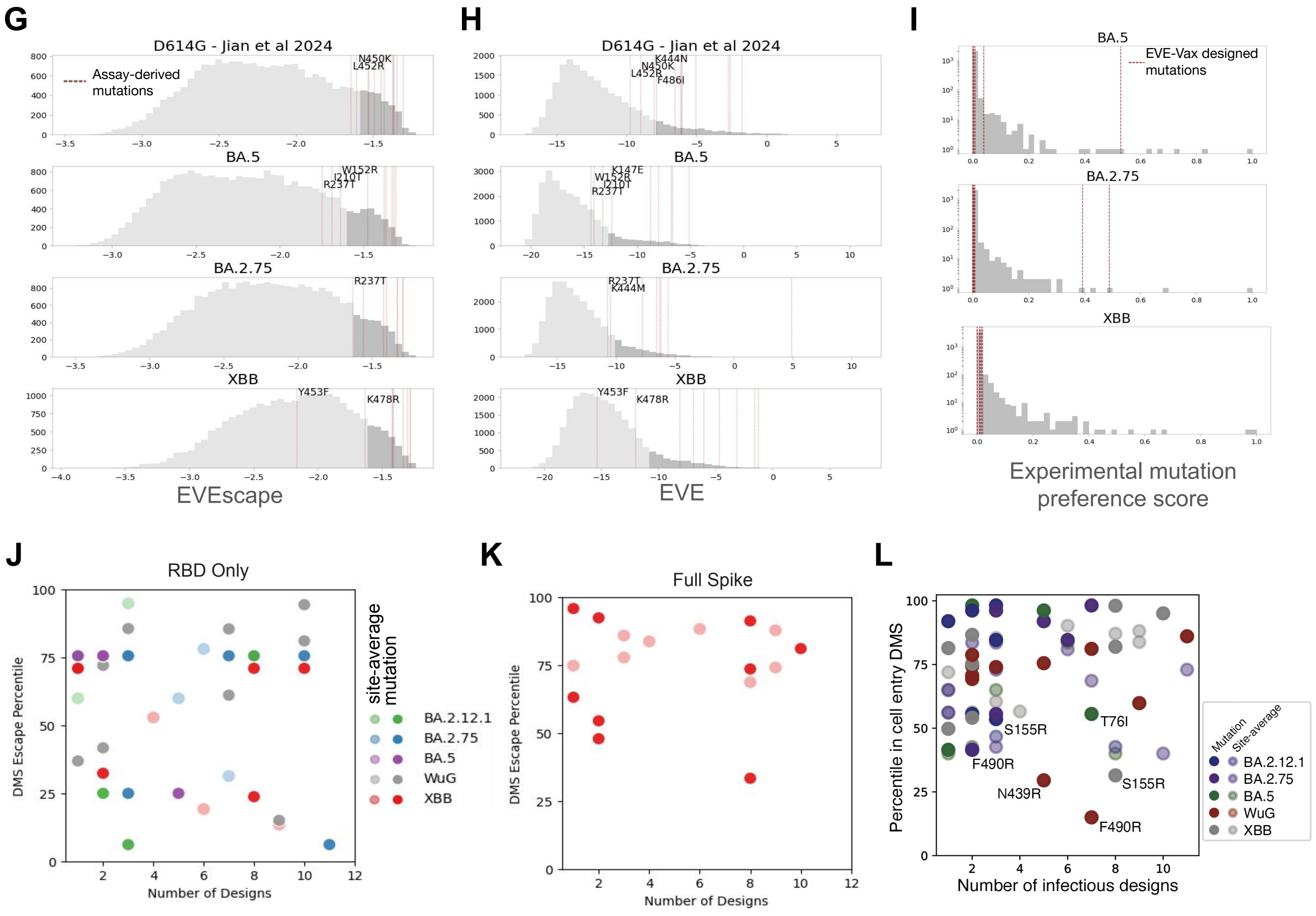

Figure S5: Computational and assay-derived designs exhibit comparable neutralization against matched sera. Related to Figure 5. **(A-D)** Fold reduction in ID50 titers for experimental or computational designs relative to parent variant in matched sera. (**A**) B.1 constructs compared to Schmidt et al [S2] B.1 designs. (**B**) BA.2.75 and (**C**) BA.5 constructs compared to Cao et al. [S3]. **(D)** XBB designs compared to Yisimayi et al. [S4] XBB.1.5 designs. **(E)** Mutated sites in computational and assay-derived designed constructs are highly frequent in GISAID [S1]. (**F**) Mutation counts across the Spike protein for all sites (left) and sites mutated at least 20 thousand times. Spike regions: All (1-1273), S1 (1-686), NTD (13-305), RBD (319-541), and RBM (437-508). **(G-H)** Mutations identified using high throughput experiments have high EVEscape and EVE scores. Dark grey: Mutation in top 10%. Vertical lines: Mutation designed using assay-driven approaches: D614G (Jian et al [S5]), BA.5 (Cao et al [S3]), BA.2.75 (Cao et al [S3]), XBB.1.5 (Yisimayi et al [S4]). **(I)** Computationally designed mutations could not have been identified using the assay-driven approach. Histogram: distribution of experimental scores used in Cao et al [S3] (BA.5 and BA.2.75) and Yisimayi et al [S4] (XBB*). Vertical lines: EVE-Vax designed mutations. **(J)** Percentile in RBD only DMS antibody escape scores of EVE-Vax designed mutations. Antibody escape scores are the maximum across hundreds of monoclonal antibodies from the respective study: Cao et al. [S3] for the B.1 (WuG) designs, Yisimayi et al. [S4] for BA.5, BA.2.12.1, and BA.2.75 rounds, and Jian et al. [S5] for XBB round. **(K)** Percentile in full Spike DMS escape measured based on escape scores from Dadonaite et al [S6]. (**L**) Percentile in DMS cell entry scores [S6] of EVE-Vax designed mutations. Many mutations with highly deleterious cell entry measurements appear successfully in multiple designs and across different backgrounds. The DMS with the closest parent variant to the round was chosen: B.1 round shown with SARS-CoV-2 Delta DMS (Dadonaite et al. [S7]), BA.5, BA.2.12.1 and BA.275 rounds shown with BA.2 DMS (Dadonaite et al. [S6]), and XBB round shown with XBB.1.5 DMS (Dadonaite et al. [S6]). Site-averaged percentiles are shown where exact designed mutation is missing from the DMS.

Supplemental references

[S1] Khare, S., Gurry, C., Freitas, L., Schultz, M.B., Bach, G., Diallo, A., Akite, N., Ho, J., Lee, R.T., Yeo, W., et al. (2021). GISAID's Role in Pandemic Response. China CDC Wkly 3, 1049–1051.

[S2] Schmidt, F., Weisblum, Y., Muecksch, F., Hoffmann, H.-H., Michailidis, E., Lorenzi, J.C.C., Mendoza, P., Rutkowska, M., Bednarski, E., Gaebler, C., et al. (2020). Measuring SARS-CoV-2 neutralizing antibody activity using pseudotyped and chimeric viruses. J. Exp. Med. 217.

[S3] Cao, Y., Jian, F., Wang, J., Yu, Y., Song, W., Yisimayi, A., Wang, J., An, R., Chen, X., Zhang, N., et al. (2023). Imprinted SARS-CoV-2 humoral immunity induces convergent Omicron RBD evolution. Nature 614, 521–529.

[S4] Yisimayi, A., Song, W., Wang, J., Jian, F., Yu, Y., Chen, X., Xu, Y., Yang, S., Niu, X., Xiao, T., et al. (2024). Repeated Omicron exposures override ancestral SARS-CoV-2 immune imprinting. Nature 625, 148–156.

[S5] Jian, F., Wang, J., Yisimayi, A., Song, W., Xu, Y., Chen, X., Niu, X., Yang, S., Yu, Y., Wang, P., et al. (2024). Evolving antibody response to SARS-CoV-2 antigenic shift from XBB to JN.1. bioRxiv, 2024.04.19.590276.

[S6] Dadonaite, B., Brown, J., McMahon, T.E., Farrell, A.G., Figgins, M.D., Asarnow, D., Stewart, C., Lee, J., Logue, J., Bedford, T., et al. (2024). Spike deep mutational scanning helps predict success of SARS-CoV-2 clades. Nature.

[S7] Dadonaite, B., Crawford, K.H.D., Radford, C.E., Farrell, A.G., Yu, T.C., Hannon, W.W., Zhou, P., Andrabi, R., Burton, D.R., Liu, L., et al. (2023). A pseudovirus system enables deep mutational scanning of the full SARS-CoV-2 spike. Cell 186, 1263–1278.e20.
